# Metabolic adaptations direct cell fate during tissue regeneration

**DOI:** 10.1038/s41586-025-09097-6

**Published:** 2025-06-11

**Authors:** Almudena Chaves-Perez, Scott E. Millman, Sudha Janaki-Raman, Yu-Jui Ho, Clemens Hinterleitner, Valentin J. A. Barthet, John P. Morris, Francisco M. Barriga, Jose Reyes, Aye Kyaw, H. Amalia Pasolli, Dana Pe’er, Craig B. Thompson, Lydia W. S. Finley, Justin R. Cross, Scott W. Lowe

**Affiliations:** 1 Cancer Biology and Genetics Program, Memorial Sloan Kettering Cancer Center, New York, NY USA; 2 Donald B. and Catherine C. Marron Cancer Metabolism Center, Memorial Sloan Kettering Cancer Center, New York, NY USA; 3 Computational and Systems Biology Program, Memorial Sloan Kettering Cancer Center, New York, NY USA; 4 Electron Microscopy Resource Center, The Rockefeller University, New York, NY USA; 5 Cell Biology Program, Memorial Sloan Kettering Cancer Center, New York, NY USA

**Keywords:** Stem-cell differentiation, Intestinal stem cells, Ulcerative colitis, Energy metabolism

## Abstract

Although cell-fate specification is generally attributed to transcriptional regulation, emerging data also indicate a role for molecules linked with intermediary metabolism. For example, α-ketoglutarate (αKG), which fuels energy production and biosynthetic pathways in the tricarboxylic acid (TCA) cycle, is also a co-factor for chromatin-modifying enzymes^1–3^. Nevertheless, whether TCA-cycle metabolites regulate cell fate during tissue homeostasis and regeneration remains unclear. Here we show that TCA-cycle enzymes are expressed in the intestine in a heterogeneous manner, with components of the αKG dehydrogenase complex^4–6^ upregulated in the absorptive lineage and downregulated in the secretory lineage. Using genetically modified mouse models and organoids, we reveal that 2-oxoglutarate dehydrogenase (OGDH), the enzymatic subunit of the αKG dehydrogenase complex, has a dual, lineage-specific role. In the absorptive lineage, OGDH is upregulated by HNF4 transcription factors to maintain the bioenergetic and biosynthetic needs of enterocytes. In the secretory lineage, OGDH is downregulated through a process that, when modelled, increases the levels of αKG and stimulates the differentiation of secretory cells. Consistent with this, in mouse models of colitis with impaired differentiation and maturation of secretory cells, inhibition of OGDH or supplementation with αKG reversed these impairments and promoted tissue healing. Hence, OGDH dependency is lineage-specific, and its regulation helps to direct cell fate, offering insights for targeted therapies in regenerative medicine.

## Abstract (web summary)

Metabolic enzymes of the tricarboxylic acid cycle, such as 2-oxoglutarate dehydrogenase, are differentially expressed in absorptive and secretory lineages, guiding cell fate establishment and offering insights for targeted regenerative therapies.

## Main

In the mammalian intestine, cells undergo a hierarchical differentiation process, which generates distinct lineages that contribute to the various cell types of the intestinal crypt^7,8^. Intestinal stem cells (ISCs), which reside at the base of the crypt, have the ability to self-renew and to generate the lineages that make up the intestinal epithelium. Stemness in ISCs is maintained by a balance between the bone morphogenic protein (BMP), Notch and WNT signalling pathway and the WNT agonist R-spondin^9–11^. As ISCs divide, daughter cells migrate to the transit-amplifying cell compartment, where they generate progenitor cells^12^. The subsequent activation of lineage-specific transcriptional programs drives the full differentiation of these progenitors into mature absorptive and secretory lineages. The absorptive lineage arises through enterocyte progenitors, which must undergo rapid expansion to form the absorptive surface in the intestine, estimated at 260–300 m^2^ in humans^9,13,14^. Commitment to the secretory lineage instead produces a smaller but diverse pool of cells, including Paneth, enteroendocrine, goblet and tuft cells. These specialized cells are involved in host defence, mucus secretion and immune modulation, all of which are essential to maintain intestinal health^7,8^. Intestinal injury can disrupt the trajectory of ISC differentiation, resulting in impaired maturation and a decrease in the number of secretory cells^15–17^. This imbalance has been suggested to contribute to the pathogenesis of inflammatory bowel diseases, including Crohn’s disease and ulcerative colitis, for which further therapeutic advances are needed^15–17^.

As cells differentiate, their metabolism often changes to support the varying bioenergetic and biosynthetic needs of different cell types^18^. For energy generation, ISCs rely mainly on glycolysis^19,20^. Differentiation into highly proliferative progenitors creates an increased dependence on oxidative phosphorylation (OXPHOS)^21–23^, whereas differentiation into the secretory lineage is associated with a reduced reliance on mitochondrial electron transport chain activity^1,22^. However, mitochondrial metabolism can also directly influence cell-state transitions through rewiring of the TCA cycle^24^ or through production of metabolites that act as key co-substrates for chromatin-modifying enzymes^2,25^.

One such metabolite is αKG^1–3^. αKG is an intermediate component of the TCA cycle, and is generated through the conversion of isocitrate by the isocitrate dehydrogenase (IDH) family. Next, αKG is converted into succinyl-CoA by OGDH, a component of the αKG dehydrogenase complex^4–6^. Besides its role in canonical TCA-cycle activities, αKG is also an obligatory co-substrate of αKG-dependent dioxygenases, a family of around 70 enzymes that are involved in a range of cellular activities^25^, including epigenetic regulation. Experimental perturbations in central-carbon metabolism that increase the αKG/succinate ratio can enhance the activity of αKG-dependent dioxygenases and bias embryonic stem (ES) cells and certain models of cancer towards differentiation^1–3^. However, how cell-fate decisions are regulated by αKG in tissues remains unclear.

## Metabolic divergence in intestinal lineages

**Fig. 1:**
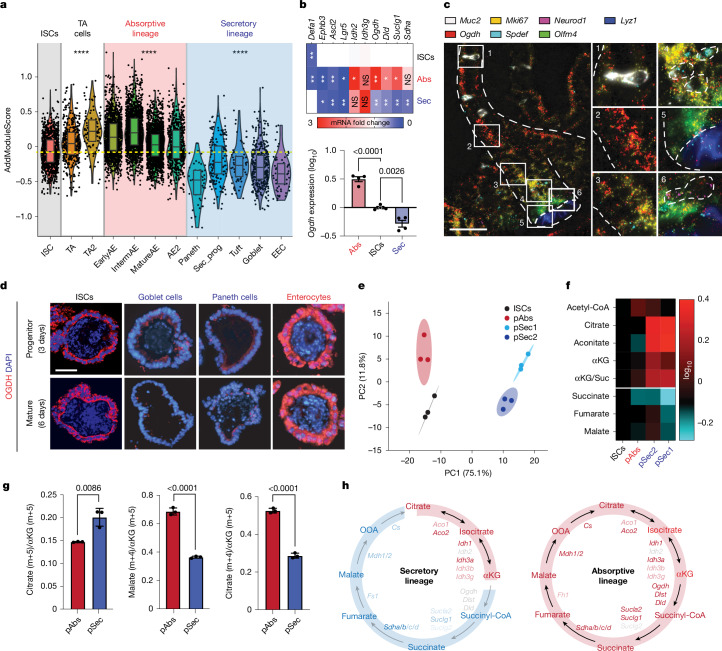
Metabolic divergence in intestinal lineages. **a**, AddModule Score showing average expression of the TCA-cycle signature across the indicated intestinal lineages in human small intestine. Each dot represents a cell. **b**, Heat map showing the transcriptional expression of lineage-specific markers and TCA-cycle enzymes in distinct intestinal cell populations by qPCR analysis. Paneth cells, ISCs and the absorptive lineage (villus fraction) were sorted from *Lgr5-GFP* reporter mice (*n* = 4). **c**, smFISH visualizing RNA of *Ogdh* and lineage-specific markers in intestinal tissue from C57Bl/6 mice. Dashed lines outline crypt and villus structures in the intestinal epithelium. Results are representative of three independent experiments. **d**, Immunofluorescence showing *Ogdh* expression in ISC-enriched organoids and organoids from the indicated lineages at two stages of maturation. Results are representative of three independent experiments. **e**, Principal component analysis (PCA) of metabolite profiles from LC–MS/MS on organoids enriched for ISCs, secretory progenitors (pSec1 (goblet cell progenitors) and pSec2 (Paneth cell progenitors)) and pAbs. **f**, Heat map depicting the levels of TCA-cycle metabolites in organoids enriched for different intestinal progenitors relative to ISC-enriched organoids. αKG/Suc represents the ratio of αKG to succinate (Suc). **g**, Ratio of corrected abundance of the indicated fractions in pSec- versus pAbs-enriched organoids after glutamine isotopologue tracing. Data are representative of two independent experiments (*n* = 10 mice). **h**, Schematic of differences in the TCA cycle between absorptive and secretory lineages. For all organoid experiments, replicates were generated by isolating and pooling crypts from five mice and plating and culturing each pool in triplicate in a separate well. This figure is adapted from our published patent (WO2024229094A1)^50^. Data are mean ± s.e.m. Statistical significance was determined by Wilcoxon test in **a** (Supplementary Table 1) and two-tailed *t*-test in **g**. Asterisks indicate statistical significance (*P < 0.05, **P < 0.01, ****P < 0.0001; NS, not significant). TA, transit amplifying; TA2, transit amplifying 2; EarlyAE, early absorptive enterocytes; AE2, absorptive enterocytes 2; Sec_prog, secretory progenitors; EEC, enteroendocrine cells. Scale bars, 10 μm (**d**), 30 μm (**c**). Source Data

**Extended Data Fig. 1:**
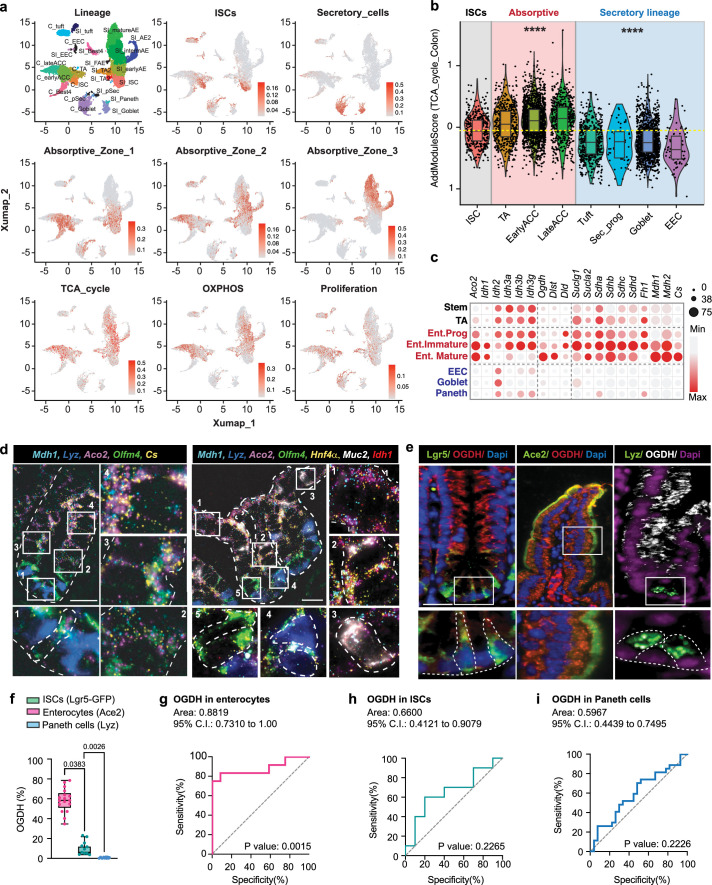
Characterization of OGDH expression in the gut. **a**, UMAP derived from publicly available scRNA-seq data demonstrating distinct transcriptional signatures in various subpopulations of human intestinal and colonic cells. **b**, AddModule Score showing average expression of the TCA-cycle signature across the indicated intestinal lineages human small intestine. Each dot represents a cell. **c**, Dot plot showing TCA-cycle enzyme gene expression across intestinal cell types from publicly available scRNA-seq data. Colour intensity indicates expression levels and dot size indicates the percentage of positive cells in each lineage. Paneth cells were sorted as GFP-, FSC-A-high and ISCs were sorted as GFP-high from *Lgr5-EGFP* reporter mice. **d**, Single-molecule in situ fluorescence (smFISH) visualizing RNAs of the indicated TCA-cycle enzymes in intestinal tissue samples from C57Bl/6 mice. Dashed lines outline crypt structure and specific lineages within the crypt. **e**, Representative immunofluorescence results from intestinal tissues from Lgr5-EGFP mice showing GFP (LGR5; marking ISCs), OGDH, Lyz (Paneth cells) and ACE2 (enterocytes). **f**, Quantification of the percentage of OGDH-expressing cells in the indicated intestinal lineages. Each dot represents a mouse (enterocytes n = 17; ISCs n = 9; Paneth cells n = 9). **g**–**i**, Receiver operating characteristic (ROC) curve analysis assessing the sensitivity and specificity of OGDH as a marker of enterocytes (**g**), ISCs (**h**) and Paneth cells (**i**). Statistical analysis: data are presented as mean ± s.e.m. The box represents the interquartile range (IQR) with the median as a central line. Whiskers extend to 1.5×IQR beyond Q1 and Q3. This figure is adapted from our published patent (WO2024229094A1)^50^. Significance was determined by Wilcoxon test in **b** (see Supplementary Table 1), one-way ANOVA followed by Tukey’s HSD test in **f** and Wilson/Brown test in **g**–**i**. Asterisks indicate statistical significance (*P < 0.05, **P < 0.01, ***P < 0.001). Source Data

To investigate how the TCA cycle influences cell-fate decisions, we used the mouse intestine as a model system for multilineage tissue differentiation and regeneration. Contrary to the notion that TCA-cycle enzymes are expressed ubiquitously, our analysis of publicly available datasets from single-cell RNA sequencing (scRNA-seq) of human intestinal and colonic mucosa^26–28^, along with quantitative PCR (qPCR) on sorted cells from an ISC reporter mouse, revealed notable heterogeneity in the expression of these enzymes. Absorptive cells, compared with ISCs, exhibited enriched expression of most TCA-cycle enzymes (Fig. 1a,b, Extended Data Fig. 1a–c and Supplementary Table 1). Conversely, the secretory lineage was characterized by a low score for the TCA-cycle gene signature (Fig. 1a,b, Extended Data Fig. 1a–c and Supplementary Table 1) and by reduced expression levels of several enzymes, starting with the αKG dehydrogenase complex, as shown by qPCR analysis in sorted cells (Fig. 1b). These results were confirmed by single-molecule fluorescence in situ hybridization (smFISH) and immunofluorescence (Fig. 1c and Extended Data Fig. 1d–i). Across lineages, differences in the expression of TCA-cycle enzymes might relate to their distinct metabolic needs and point towards potential differences in αKG abundance during intestinal differentiation.

**Extended Data Fig. 2:**
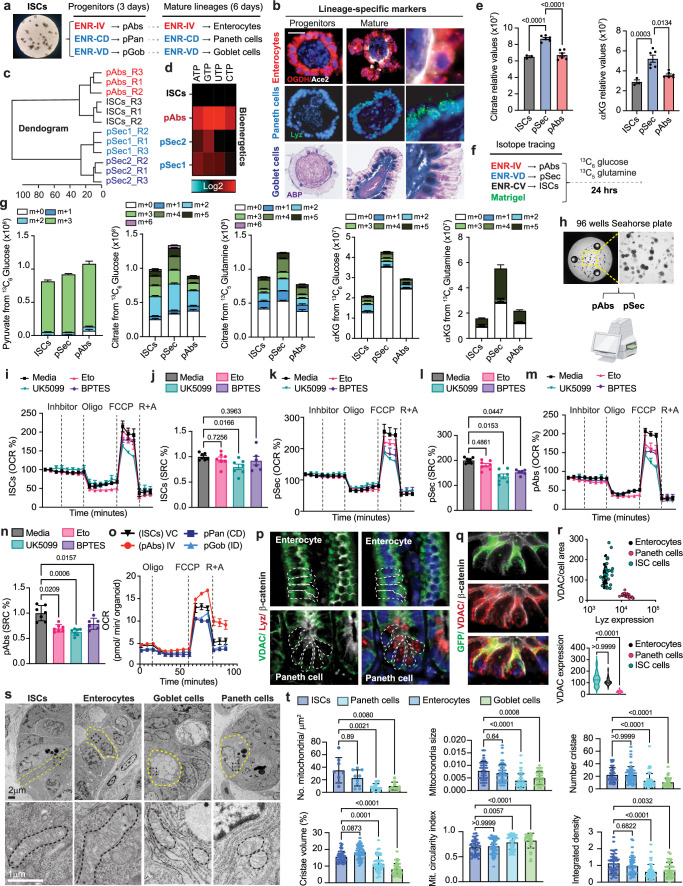
Mitochondrial activity and TCA-cycle characterization in intestinal lineages. **a**, Strategy for metabolic experiments using various ENR media to culture and differentiate intestinal progenitor organoids. **b**, Immunofluorescence and ABP staining in progenitor-enriched and mature organoids: ACE2 marks enterocytes; Lyz marks Paneth cells; ABP marks goblet cells. **c**, Hierarchical clustering dendrogram of LC–MS metabolites from intestinal organoids. **d**, Heat map of bioenergetic metabolites in intestinal progenitors relative to ISCs. **e**, LC–MS analysis of steady-state αKG and citrate levels in organoid-derived intestinal lineages. Each dot represents a replicate, generated by pooling crypts from five mice and culturing in a separate well. Data are representative of two independent experiments (n = 10 mice). **f**, Isotopologue tracing in organoids. **g**, LC–MS analysis of αKG, citrate and pyruvate labelling from ^13^C_6_-glucose or ^13^C_5_-glutamine. Levels are shown as peak area x fractional labelling (n = 3/condition). Results are representative of two independent experiments (n = 10 mice). **h**, Micrograph of organoids in a Seahorse plate for MitoStress and Substrate oxidation assays. Created in BioRender. Chaves-Perez, A. (2025) https://BioRender.com/0m4ci27. **i**–**n**, Oxygen consumption rate (OCR) (**i,k,m,n**) and spare respiratory capacity (SRC) (**j,l**) from substrate oxidation assays in organoid-derived intestinal lineages (n = 5). Inhibitors: Etomoxir (Eto; fatty acid oxidation), UK5099 (mitochondrial pyruvate carrier), BPTES (glutamine oxidation). **o**, OCR from MitoStress analysis in progenitor lineages. For **i**–**o**, data represent two independent experiments (n ≥ 3 mice/lineage). **p**,**q**, Immunofluorescence in intestinal tissues from C57Bl/6 (**p**) and *Lgr5-EGFP* (**q**) mice, showing lysozyme (Paneth), VDAC (mitochondria), β-catenin (membrane), and GFP (ISCs). Dashed lines delineate cells of a specific lineage. **r**, Quantification of VDAC intensity in ISCs, enterocytes and Paneth cells. Each dot represents an independent crypt (n = 4 mice). **s**, Electron microscopy images of mitochondria in various intestinal lineages from C57Bl/6 mice. Dashed lines delineate cells of a specific lineage. **t**, Quantification of multiple mitochondria parameters from **s**. Each dot represents a cell for the specified lineage. This figure is adapted from our published patent (WO2024229094A1)^50^. Statistical analysis: data are presented as mean ± s.e.m. Statistical significance was determined by one-way ANOVA followed by Tukey’s test in **e**,**j**,**l**,**n**,**r** (bottom graph) and **t**. OCRs were analysed using a two-way ANOVA followed by Tukey’s HSD test. Asterisks indicate statistical significance (*P < 0.05, **P < 0.01, ***P < 0.001). Abbreviations: pAbs=absorptive progenitors; C = CHIR2099; D = DAPT; ENR = EGF/Noggin/R-spondin; I = IWP2; pSec1=goblet progenitors; pSec2=Paneth progenitors; R + A=Rotenone + Antimycin A; V=Valproic acid. Source Data

To evaluate this functionally, we used an organoid model in which intestinal differentiation can be controlled by culture conditions^29^ (Extended Data Fig. 2a). After confirming that this system captures the in vivo heterogeneity of OGDH expression (Fig. 1d and Extended Data Fig. 2b), we determined the composition of metabolites in organoids enriched for secretory progenitors (pSec1 and pSec2; goblet cell progenitors and Paneth cell progenitors, respectively), absorptive progenitors (pAbs; enterocyte progenitors) and ISCs by applying ion-pair liquid chromatography coupled with tandem mass spectrometry (LC–MS/MS). Through these analyses, we identified 299 metabolites with differential abundance across lineages (Fig. 1e and Extended Data Fig. 2c). Organoids enriched for pAbs exhibited a relative increase in the abundance of metabolites implicated in energy production (ATP) and biosynthetic processes (GDP, GTP, dUTP and dXMP) (Fig. 1f and Extended Data Fig. 2d). By contrast, pSec1- and pSec2-enriched organoids showed increased levels of citrate, aconitate and αKG (around 50% higher compared with ISCs and around 40% higher compared with pAbs), but reduced levels of downstream TCA-cycle intermediates (Fig. 1f and Extended Data Fig. 2e). As a result, pSec1 and pSec2 showed an increase in the αKG/succinate ratio, whereas pAbs did not (Fig. 1f). Furthermore, these secretory progenitors had less ATP (Extended Data Fig. 2d), albeit enough to support the differentiation and viability of secretory cells. These data suggest that each lineage has distinct metabolic requirements.

We next further examined the source of TCA-cycle metabolites. Carbon tracing experiments using ^13^C_5_ glutamine and ^13^C_6_ glucose (Extended Data Fig. 2f) showed that, compared with pAbs organoids, pSec organoids exhibited a relative decrease in total metabolite levels in the oxidative synthesis of malate (Extended Data Fig. 2g). This is consistent with their lower levels of OGDH expression, and implies reduced TCA-cycle activity. Additionally, pSec lineages showed an increase in total αKG, as well as increases in αKG fractional labelling derived from glucose and glutamine; this was accompanied by reduced oxidative carboxylation, as evidenced by a decrease in the malate (m+4)/aKG (m+5) and citriate (m+4)/aKG (m+5) ratio, and enhanced reductive carboxylation, as evidenced by increases in the citrate (m+5)/αKG (m+5) ratio derived from ^13^C_5_ glutamine tracing (Fig. 1g and Extended Data Fig. 2e,g).

**Extended Data Fig. 3:**
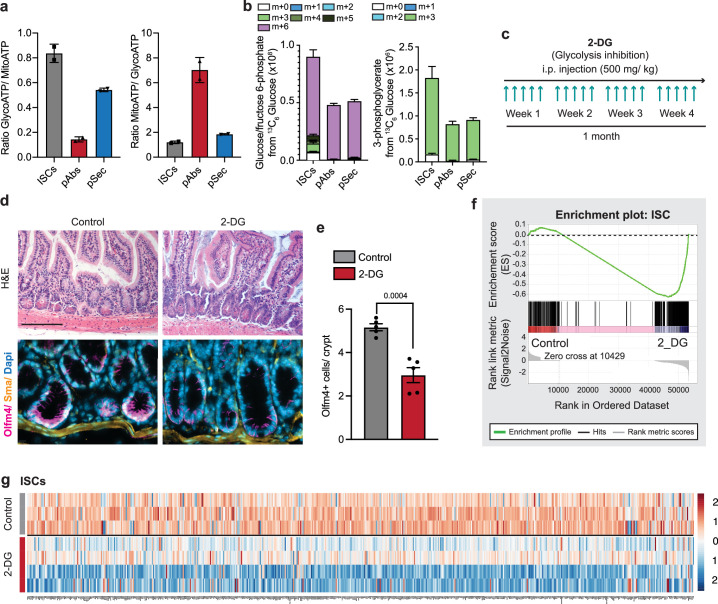
Effect of glycolysis inhibition in ISCs. **a**, ATP rate assay in ISCs- and pAbs-enriched organoids showing the production of ATP through OXPHOS (MitoATP) or glycolysis (GlycoATP) in organoids. Each dot represents an independent experiment where five mice (n = 5) were pooled, and each lineage in each experiment was plated as 8 technical replicates. **b**, LC–MS was used to determine the steady-state level and fraction of glucose/ fructose 6-phosphate and 3-phosphoglycerate from ^13^C_6_-glucose. Glucose/ fructose 6-phosphate and 3-phosphoglycerate levels are shown as peak area in arbitrary units (a.u.) x fractional percentage of labelling with each tracer (n = 3/condition). Results are representative of 2 independent experiments. **c**, Experimental design for glycolysis inhibition in C57Bl/6 mice. **d**, H&E staining and immunofluorescence of ISCs (OLFM4^+^ cells) of control and 2-deoxyglucose (2-DG)-treated mice after one month of treatment. **e**, Quantification of mean OLFM4^+^ cells per crypt from (**c**). Each dot represents one mouse (n = 5). At least 30 crypts were analysed per mouse. **f**, Gene set enrichment analysis (GSEA) showing downregulation of the ISC gene signature in intestinal tissue from 2-DG–treated mice compared with control mice. **g**, Heat map illustrating transcription of genes in the ISC signature in intestinal tissue from control and 2-DG treated mice. Each row represents an individual mouse. This figure is adapted from our published patent (WO2024229094A1)^50^. Statistical significance: data represent mean ± s.e.m. Statistical significance was determined by two-tailed t-test in **e**. In each panel, asterisks indicate statistical significance (***P < 0.001). Source Data

These differences in TCA-cycle gene expression and associated metabolite abundance suggest that each lineage differentially regulates TCA-cycle output to achieve distinct metabolic requirements. Substrate oxidation assays revealed that enterocytic progenitors used glutamine and fatty acids as alternative carbon sources to glucose for energy production, whereas secretory progenitors depended strongly on glutamine, a crucial precursor for αKG (Extended Data Fig. 2h–n). Furthermore, secretory progenitors had a lower spare respiratory capacity than ISCs and pAbs (Extended Data Fig. 2o). Whereas the mitochondria of ISCs and absorptive cells had dense cores and tightly packed cristae, the mitochondria of secretory cells were less abundant and exhibited a more relaxed morphology, characterized by larger and more spread-out cores, along with dispersed cristae (Extended Data Fig. 2p–t). These changes were also associated with functional effects on ISCs, because inhibiting glycolysis with the glucose analogue 2-deoxy-d-glucose (2-DG) reduced stemness in mouse intestines^19,20^ (Extended Data Fig. 3a–g). These findings suggest that ICS differentiation involves distinct metabolic transitions, with increased mitochondrial activity in the absorptive lineage and reduced OXPHOS in the secretory lineage. In addition, downregulation of OGDH facilitates a higher αKG/succinate ratio during secretory-lineage specification (Fig. 1h).

## Differential role of OGDH in cell fate

**Fig. 2:**
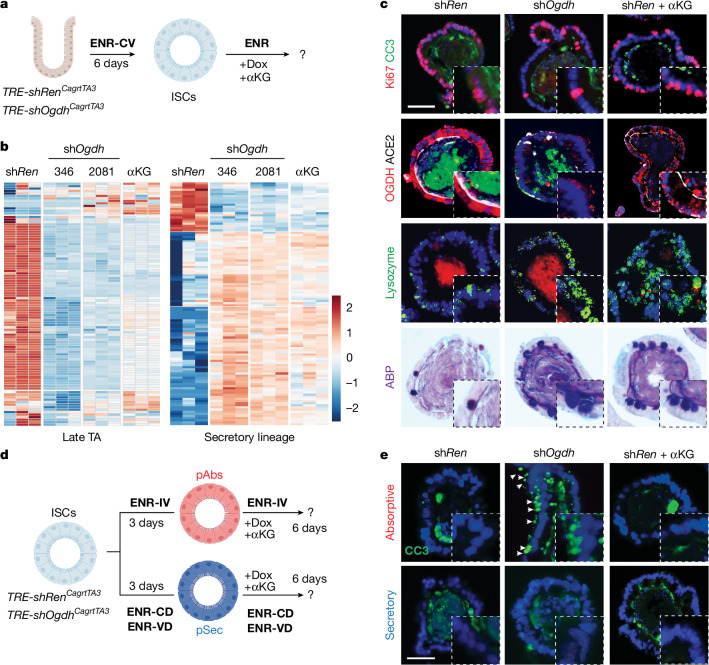
Differential role of OGDH in cell fate. **a**, Diagram of *Ogdh* suppression experiments in ISC-enriched organoids derived from *TRE*-*shRen*^*Cag-rtTA3*^, *TRE*-*shOgdh*^*Cag-rtTA3*^ or wild-type mice. ISC-enriched organoids were grown in ENR-CV medium (C, CHIR2099; ENR, EGF, Noggin and R-spondin; V, valproic acid) for six days, then treated with doxycycline (Dox) (*TRE*-*shRen*^*Cag-rtTA3*^ and *TRE*-*shOgdh*^*Cag-rtTA3*^) or DM-αKG (wild type) while switching to ENR medium to facilitate differentiation into all intestinal lineages. Created in BioRender. Chaves-perez, A. (2025) (https://BioRender.com/dimozin). **b**, Heat map showing early changes in late-TA and secretory-lineage signatures from RNA-seq analysis of organoids from *TRE*-*shRen*^*Cag-rtTA3*^ and *TRE*-*shOgdh*^*Cag-rtTA3*^ mice treated with doxycycline or DM-αKG for 72 h. Two different hairpins were used for *TRE*-*shOgdh*^*Cag-rtTA3*^ mice (346 and 2081). Each column represents organoids derived from three mice. **c**, Immunofluorescence and staining in organoids from **b**, showing cell proliferation (Ki67), cell death (cleaved caspase 3; CC3) and lineage-specific markers (ACE2, enterocytes; ABP, goblet cells; lysozyme, Paneth cells) after six days of the indicated treatments. **d**, Experimental schematic for *Ogdh* suppression and exogenous αKG supplementation studies in progenitor-enriched organoids. ISC-enriched organoids were cultured for six days in ENR-CV medium, then differentiated for three days in the pertinent lineage-specific medium (C, CHIR2099; D, DAPT; ENR, EGF, Noggin and R-spondin; I, IWP2; V, valproic acid). Subsequently, they were treated with either doxycycline (*TRE*-*shRen*^*Cag-rtTA3*^ and *TRE*-*shOgdh*^*Cag-rtTA3*^ organoids) or DM-αKG (*TRE*-*shRen*^*Cag-rtTA3*^ organoids). Organoids were cultured in the appropriate lineage-specific differentiation medium for six days to direct differentiation into the desired cell lineage. Created in BioRender. Chaves-perez, A. (2025) (https://BioRender.com/dimozin). **e**, Immunofluorescence in progenitor-enriched control (*TRE*-*shRen*^*Cag-rtTA3*^), OGDH-depleted (*TRE*-*shOgdh*^*Cag-rtTA3*^) or αKG-treated (*TRE*-*shRen*^*Cag-rtTA3*^) organoids showing cell death (cleaved caspase 3) after eight days in culture. This figure is adapted from our published patent (WO2024229094A1)^50^. Scale bars, 10 μm (**c**,**e**).

**Extended Data Fig. 4:**
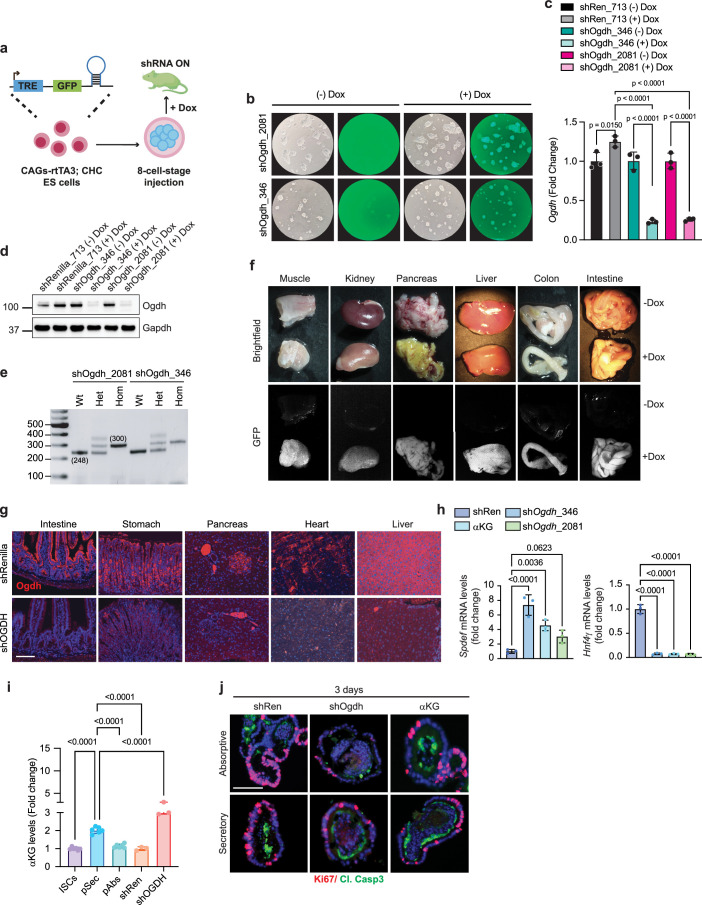
Generation of an inducible *Ogdh*-knockdown model. **a**, Schema of engineering of *TRE*-*shOgdh* knockdown mouse model via the ‘speedy-mouse’ method (see Methods). Created in BioRender. Chaves-Perez, A. (2025) https://BioRender.com/8xmcqsn. **b**, GFP induction in *shOgdh_346* and *shOgdh_2081* mouse ES cells after a 48-h doxycycline treatment. **c**, qPCR quantification of *Ogdh* mRNA in shRenilla.713, *shOgdh_346* and *shOgdh_2081* mouse ES cells after the 48-h doxycycline treatment. Each dot represents an independent ES cell line (n = 3). **d**, Western analysis of mouse ES cells used to generate the mouse model, after the 48-hour doxycycline treatment. **e**, PCR showing wild-type genotype (248 bp) and knock-in genotype (300 bp). **f**, Representative bright-field and GFP fluorescence images of various organs from *TRE*-*shOgdh*^*Cag-rtTA3*^ mice fed a normal or doxycycline (Dox) diet for 6 days. **g**, Representative immunofluorescent images showing OGDH in the indicated tissues on day 3 of doxycycline treatment in *TRE*-*shOgdh*^*rtTA3*^ and *TRE*-*shRenilla*^*Cag-rtTA3*^ mice (n = 5). **h**, qPCR analysis of *Spdef* and *Hnf4g* mRNA expression in organoids derived from shRen^Cag-rtTA3^ and *TRE*-shOgdh^Cag-rtTA3^ mice, treated with either doxycycline or DM-αKG for 72 h. Each dot represents a different mouse (n = 3 mice). **i**, LC–MS analysis of αKG levels, depicted as fold changes in sh*Ogdh* versus pSec organoids. **j**, Representative immunofluorescent images of progenitor-enriched organoids from *TRE*-*shRen*^*Cag-rtTA3*^, *TRE*-*shOgdh*^*Cag-rtTA3*^, and DM-αKG-treated organoids, visualizing cell proliferation (Ki67) and cell death (Cl. Casp3) on day 3 of treatment. This figure is adapted from our published patent (WO2024229094A1)^50^. Statistical significance: data represent mean ± s.e.m. Statistical significance was determined using one-way ANOVA followed by Tukey’s HSD test in **c**,**h**,**i**. Asterisks denote significance (*P < 0.05, **P < 0.01, ***P < 0.001). Source Data

To investigate the role of OGDH in intestinal differentiation, we generated transgenic mice (*TRE*-*shOgdh*) in which *Ogdh* could be silenced in intestinal organoids or throughout the mouse using a doxycycline-inducible short hairpin RNA (shRNA) transgenic model^30–32^ (Extended Data Fig. 4a). Inducible and robust GFP induction coupled with potent *Ogdh* suppression was confirmed in ES cells (Extended Data Fig. 4b–e). After establishing germline strains, *TRE-shOgdh* mice were crossed with *CAGs-rtTA3* transgenic mice to allow systemic and inducible *Ogdh* suppression^33^ (Extended Data Fig. 4f,g). Next, we derived organoids from *TRE-shOgdh*^*Cag-rtTA3*^ mice and control *TRE-shRen*^*Cag-rtTA3*^ mice (expressing a neutral shRNA that targets Renilla luciferase). Organoids cultured in ISC-enriching medium or under differentiation conditions were treated with doxycycline to suppress OGDH and subsequently analysed for changes in proliferation, lineage specification and cell death. Similar phenotypes were observed with two distinct *Ogdh*-targeting shRNAs (Fig. 2b and Extended Data Fig. 4). Because *Ogdh* suppression perturbs TCA-cycle activity and increases αKG abundance, to distinguish between these effects we performed complementary experiments in wild-type organoids that we treated with cell-permeable dimethyl (DM)-αKG.

*Ogdh* knockdown or DM-αKG supplementation led to lineage-specific variations in proliferation, differentiation and cell viability. In organoids enriched for ISCs, *Ogdh* suppression promoted a shift towards commitment to the secretory lineage, as evidenced by increased lysozyme expression (characteristic of Paneth cells) and Alcian Blue–PAS (ABP) staining (specific to Goblet cells). Notably, DM-αKG supplementation in ISC-enriched organoids had a similar effect (Fig. 2a–c and Extended Data Fig. 4h). Note that the absolute levels of αKG in pSec progenitors were comparable with those achieved by OGDH depletion (Extended Data Fig. 4i). No cell death was detected after either *Ogdh* suppression or αKG addition (Fig. 2c), consistent with our results (Extended Data Fig. 3a–g) and with previous studies showing that ISCs rely on glycolysis for ATP generation^19,20^. In pSec-enriched organoids, neither OGDH depletion nor exogenous αKG affected proliferation or death (Fig. 2d,e and Extended Data Fig. 4j), aligning with the inherently low *Ogdh* expression and reduced mitochondrial reliance of the secretory lineage^19^. By contrast, *Ogdh* knockdown—but not αKG supplementation—markedly impaired the proliferation of pAbs by day 3 and induced considerable levels of cell death by day 8 (Fig. 2d,e and Extended Data Fig. 4j). These results support the idea that downregulation of OGDH increases αKG, promoting secretory differentiation of ISCs, whereas upregulation of OGDH (and other TCA enzymes) is crucial for maintaining enterocyte function.

## OGDH-driven lineage-specific mechanisms

**Fig. 3:**
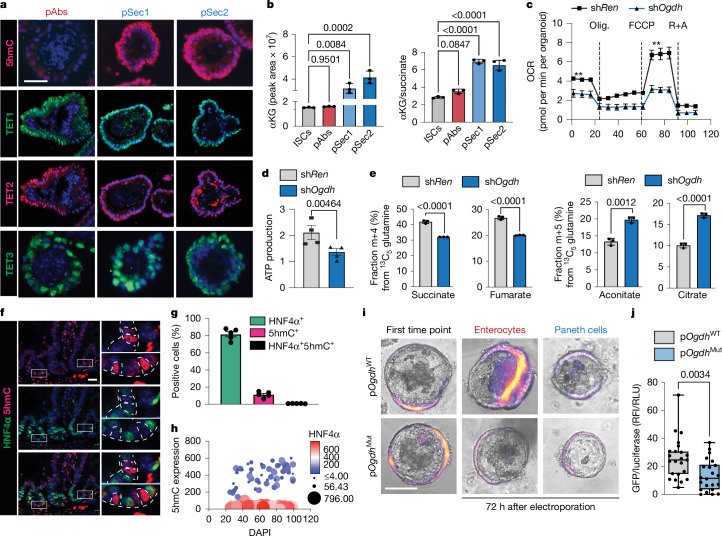
Metabolic and epigenetic divergence in intestinal progenitors. **a**, Immunofluorescence in C57Bl/6 progenitor-enriched organoids showing TET and 5hmC expression. **b**, Steady-state levels of αKG and αKG/succinate ratio by LC–MS in ISC-enriched and progenitor-enriched organoids. Each dot represents a replicate, generated by isolating and pooling crypts from five mice and plating each in triplicate in a separate well. Data are representative of two independent experiments (*n* = 10 mice). **c**, Oxygen consumption rate (OCR) in organoids derived from *TRE*-*shRen*^*Cag-rtTA3*^ or *TRE*-*shOgdh*^*Cag-rtTA3*^ mice (*n* = 4 for sh*Ren* and *n* = 4 for sh*Ogdh*). Olig., oligomycin; R+A, rotenone and antimycin. **d**, ATP production in *TRE*-*shRen*^*Cag-rtTA3*^ or *TRE*-*shOgdh*^*Cag-rtTA3*^ mice as measured on a Seahorse instrument (*n* = 4). Each dot represents one mouse. **e**, Succinate, fumarate, aconitate and citrate shown as fractional labelling with ^13^C_5_-glutamine (*n* = 3 per condition) in organoids from *TRE*-*shOgdh*^*Cag-rtTA3*^ mice, with or without doxycycline treatment for 72 h. Each dot represents a replicate, generated as explained in **b**. Data are representative of two independent experiments (*n* = 10). **f**, Immunofluorescence for HNF4α and 5hmC in crypts in tissue sections from C57Bl/6 mice. Dashed lines outline 5hmC^+^HNF4α^−^ cells. Scale bar, 20 μm. **g**, Lack of colocalization between HNF4α and 5hmC in tissues derived from **f**. Each dot represents one mouse (*n* = 5). **h**, 5hmC and HNF4α expression in individual cells from tissue sections obtained from **f**. Each dot represents one cell (*n* = 5 mice). **i**,**j**, Expression of Turbo–GFP reporter in the indicated intestinal lineages (**i**) and GFP/luciferase ratio in enterocytes at 72 h (**j**) electroporated with a reporter construct containing either wild-type or *Hnf4a*-mutant binding sites in the *Ogdh* promoter (*n* = 3 mice; 8 wells per mouse). RFI, relative fluorescence intensity; RLU, relative luminescence units. The box represents the interquartile range (IQR) with the median as a central line. Whiskers extend to 1.5 × IQR beyond Q1 and Q3. This figure is adapted from our published patent (WO2024229094A1)^50^. Data are mean ± s.e.m. Statistical significance was determined by one-way ANOVA followed by Tukey’s honestly significant difference (HSD) test in **b**,**g**, two-tailed *t*-test in **d**,**e**,**j** and two-way ANOVA followed by Tukey’s HSD test in **c**. Asterisks indicate statistical significance (**P < 0.01). Scale bars, 10 μm (**a**,**i**), 50 μm (**f**). Source Data

**Extended Data Fig. 5:**
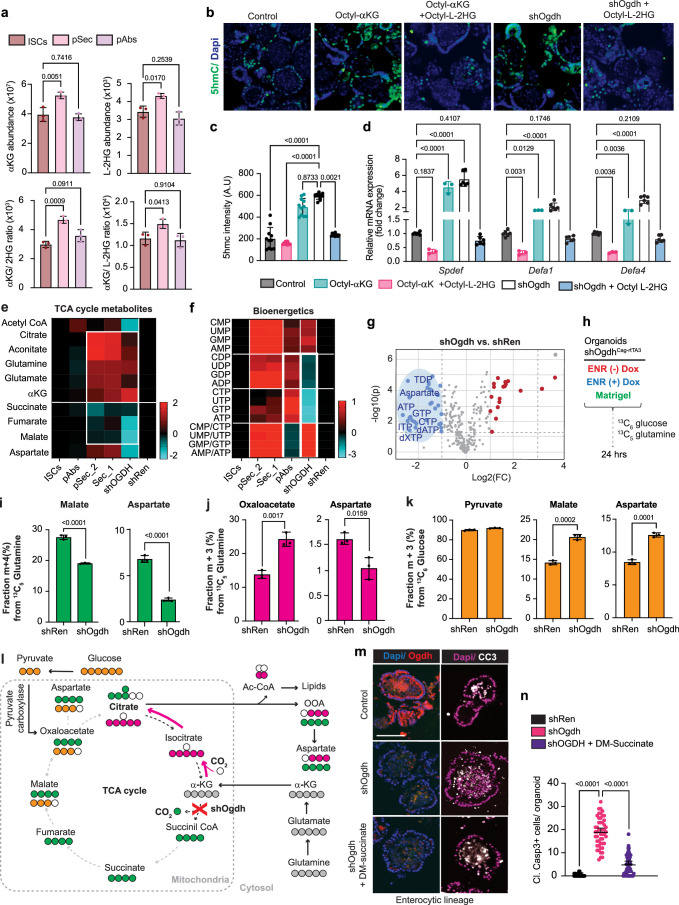
Metabolic analysis after OGDH downregulation. **a**, αKG and L-2HG abundance, and αKG:2HG and αKG:L-2HG ratio in progenitor- and ISCs-enriched organoids. Each dot represents an independent replicate (n = 3). **b**, Immunofluorescence of 5hmC in organoids derived from C57Bl/6 mice or *TRE*-*shOgdh*^*Cag-rtTA3*^ mice and treated with octyl-αKG or octyl-L-2HG for 4 days. **c**, Quantification of 5hmC levels in the organoids from **b**. Each dot represents a different mouse (n ≥ 3). **d**, qPCR for the organoids from **b**, depicting RNA expression of the indicated secretory markers. Each dot represents a different mouse (n ≥ 3). **e**,**f**, Metabolite abundance in the indicated intestinal progenitors relative to ISCs and in *TRE*-*shOgdh*^*Cag-rtTA3*^ organoids relative to *TRE*-*shRen*^*Cag-rtTA3*^ organoids. **e**, TCA-cycle metabolites. **f**, Bioenergetics. **g**, Volcano plot showing the differentially abundant metabolites when OGDH is depleted in organoids, highlighting differences in metabolite levels related to bioenergetics between the two lineages. **h**, Experimental design for isotopologue tracing in OGDH-depleted organoids. **i**–**k**, Labelled fraction of the indicated metabolites obtained from ^13^C_6_-glucose or ^13^C_5_-glutamine in OGDH-depleted organoids. Data represent three independent replicates. To generate a replicate, crypts from 5 different mice were isolated, pooled, and then plated in triplicate in separate wells for the individual replicates. **l**, Schematic representation of carbon flux in OGDH-depleted organoids. **m**, Immunofluorescence showing OGDH and cell death (cleaved Casp3; CC3) and OGDH-depleted organoids derived from *TRE*-*shRen*^*Cag-rtTA3*^ (control) or *TRE*-*shOgdh*^*Cag-rtTA3*^ mice (n = 5) treated with DM-succinate for 8 days. **n**, Number of Cl. Casp3+ cells per organoid from **m**. Each dot represents an organoid. This figure is adapted from our published patent (WO2024229094A1)^50^. Statistical analysis: Data represent mean ± s.e.m. Statistical significance was determined using one-way ANOVA followed by Tukey’s HSD test in **a**,**c**,**d**,**n**, two-tailed *t*-test in **i**–**k**. Asterisks denote statistical significance (*P < 0.05, **P < 0.01, ***P < 0.001). Source Data

αKG might promote secretory-lineage differentiation by activating key αKG-dependent dioxygenases, such as ten-eleven translocated (TET) enzymes, which convert 5-methylcytosine (5mC) to 5-hydroxymethylcytosine (5hmC), thereby reversing DNA methylation and influencing cell fate^2,25^. Although the levels of TET1–TET3 were similar in absorptive and secretory progenitor organoids, immunofluorescence analysis revealed substantially higher levels of 5hmC in secretory progenitors, with inherently increased αKG (Fig. 3a,b). Moreover, secretory-enriched organoids exhibited an increased ratio of αKG to L-2-hydroxyglutarate (L-2HG), a competitive inhibitor^34,35^ of αKG-dependent dioxygenases (Extended Data Fig. 5a). Exposing ISC-enriched organoids to octyl-L-2HG reduced the levels of 5hmC and downregulated the expression of secretory markers (*Spdef*, *Defa1* and *Defa4*), compared with octyl-αKG, another cell-permeable αKG analogue (Extended Data Fig. 5b–d). Despite increased levels of αKG in OGDH-depleted organoids, exposure to octyl-L-2HG decreased 5hmC levels and secretory marker expression (Extended Data Fig. 5b–d). Although further studies are needed to elucidate the mechanistic role of αKG-dependent dioxygenases in cell-fate specification, the above findings support a model in which αKG promotes secretory-lineage differentiation through αKG-dependent dioxygenase activation.

Knockdown of *Ogdh* in pAbs led to proliferative arrest and cell death (Fig. 2c,e), which might indicate a catastrophic metabolic decline in the enterocytic lineage owing to a biosynthetic deficit. In assays to assess mitochondrial function, suppression of OGDH compromised ATP production, basal respiration and spare respiratory capacity (Fig. 3c,d), and broadly reduced TCA intermediates and derivatives, particularly fumarate, malate and aspartate (Extended Data Fig. 5e). In addition, OGDH-depleted organoids exhibited increased ratios of AMP to ATP and of other monophosphates to triphosphates (Extended Data Fig. 5f,g), a state that can induce cell-cycle arrest and lead to cell death^36–38^. In fact, carbon tracing studies revealed that *Ogdh* suppression reduced the forward, oxidative movement of carbons from ^13^C_5_ glutamine through the TCA cycle and increased reductive carboxylation, both absolutely and relative to forward glutamine flux (Fig. 3e and Extended Data Fig. 5h–j). Moreover, the contribution of m+3 carbon from ^13^C_6_ glucose to TCA-cycle intermediates was increased, implying increased pyruvate carboxylase activity, which catalyses the carboxylation of pyruvate to oxaloacetate (Extended Data Fig. 5k). These compensatory effects were unable to maintain the bioenergetic and biosynthetic needs of the absorptive lineage (Extended Data Fig. 5l), and, accordingly, supplementing pAbs-enriched organoids with DM-succinate reduced cell death after *Ogdh* suppression (Extended Data Fig. 5m,n). Collectively, these findings highlight the lineage-specific functional consequences of TCA-cycle perturbation in the intestinal epithelium.

## HNF4 regulates *Ogdh* in enterocytes

**Extended Data Fig. 6:**
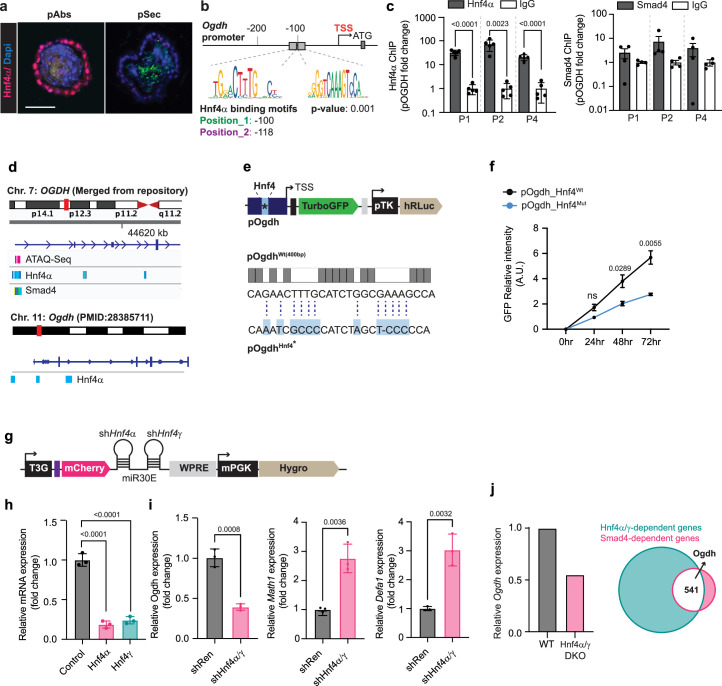
HNF4 regulates *Ogdh* expression in enterocytes. **a**, Immunofluorescent visualization of HNF4α in progenitor-enriched organoids derived from C57Bl/6 mice. The green channel was used for background. **b**, Schematic of potential binding sites for HNF4α in the *Ogdh* promoter. Positions are indicated relative to the transcriptional start site (TSS). The *P* value applies to both potential binding sites. **c**, HNF4α ChIP in isolated crypts from C57Bl/6 mice (n ≥ 4). DNA was immunoprecipitated using antibodies for HNF4α (positive binding) and SMAD4 (member of the same complex but lacking DNA-binding domains), then qPCR for the *Ogdh* promoter was run using 3 different primer pairs. Each dot represents a different mouse. **d**, HNF4α ChIP–seq data from published datasets^40^ showing HNF4 binding to the *Ogdh* promoter in human intestinal samples and crypts derived from C57Bl/6 mice. **e**, Design and sequence of reporter vectors containing wild-type or mutated HNF4-binding sites in the *Ogdh* promoter. **f**, Relative GFP intensity in colon cancer cell lines with wild-type or mutated HNF4-binding sites in the *Ogdh* promoter. Data represent three independent experiments. **g**, Design for generating an inducible vector with double hairpins to downregulate HNF4α and HNF4γ in mouse-derived organoids. **h**,**i**, qPCR for *Ogdh* (**h**) and the secretory markers *Math1* and *Defa1* (**i**) after *Hnf4a* and *Hnf4g* downregulation in ISC-enriched organoids. Data represent three independent replicates. To generate a replicate, crypts from 5 different mice were isolated, pooled, and then plated. Each replicate was independently transduced with the lentiviral construct and cultured in a separate well. **j**, *Ogdh* expression in intestinal crypts derived from Hnf4α/γ^DKO^ mice, along with a Venn diagram showing the overlap between HNF4α/γ-dependent genes and SMAD4-dependent genes, derived from published knockout studies. This figure is adapted from our published patent (WO2024229094A1)^50^. Statistical analysis: Data represent mean ± s.e.m. Statistical significance was determined using two-tailed t-test in **c**,**i**, one-way ANOVA followed by Tukey’s HSD test in **h** and two-way ANOVA followed by Tukey’s HSD test in **f**. Asterisks denote statistical significance (*P < 0.05, **P < 0.01, ***P < 0.001). Source Data

Although the regulation of TCA-cycle output across lineages is likely to be multifactorial, the transcriptional variability of TCA-cycle enzymes suggests that lineage-defining transcription factors have a role in their regulation. The HNF4 family, master regulators of enterocyte lineage specification^39^, are expressed at significantly higher levels in pAbs than in pSec1 and pSec2 progenitor organoids (Extended Data Fig. 6a). HNF4α-positive enterocytes were mutually exclusive with 5hmC-positive cells, which, as demonstrated above, is enriched in the secretory lineage (Fig. 3f–h). In support of a direct role for HNF4 in OGDH regulation in enterocytes, we identified HNF4-binding sites in the *OGDH* promoter, which were occupied by HNF4 in both mouse and human intestine, as shown by chromatin immunoprecipitation (ChIP) and analysis of publicly available ChIP–seq datasets^40^ (Extended Data Fig. 6b–d).

To assess the role of HNF4 in regulating *Ogdh*, we first examined the contribution of the HNF4-binding site to *Ogdh* transcription using *Ogdh* promoter–GFP reporter constructs with wild-type or mutated HNF4-binding sites in ISC-enriched organoids, and subjected them to differentiating conditions (Extended Data Fig. 6e). Reporter expression increased in differentiated enterocytes and declined during secretory lineage differentiation. Increases in GFP were dependent on the HNF4-binding sites (Fig. 3i,j and Extended Data Fig. 6f).

To determine whether HNF4 factors are required for *Ogdh* expression during the differentiation of the absorptive lineage, we simultaneously knocked down *Hnf4a* and *Hnf4g*, which have redundant roles in enterocytic specification^39^. This perturbation reduced the levels of *Ogdh* mRNA and skewed the differentiation of ISCs towards the secretory lineage (Extended Data Fig. 6g–i). Analysis of RNA-seq data obtained from the intestines of *Hnf4a*/*Hnf4g* double-knockout mice^39^ confirmed that HNF4 is required for robust *Ogdh* expression (Extended Data Fig. 6j). These findings show that lineage-directing transcription factors such as HNF4 regulate metabolic enzymes such as OGDH, and that this interaction is essential for enterocyte energy balance and survival.

## Role of OGDH in gut homeostasis in vivo

**Extended Data Fig. 7:**
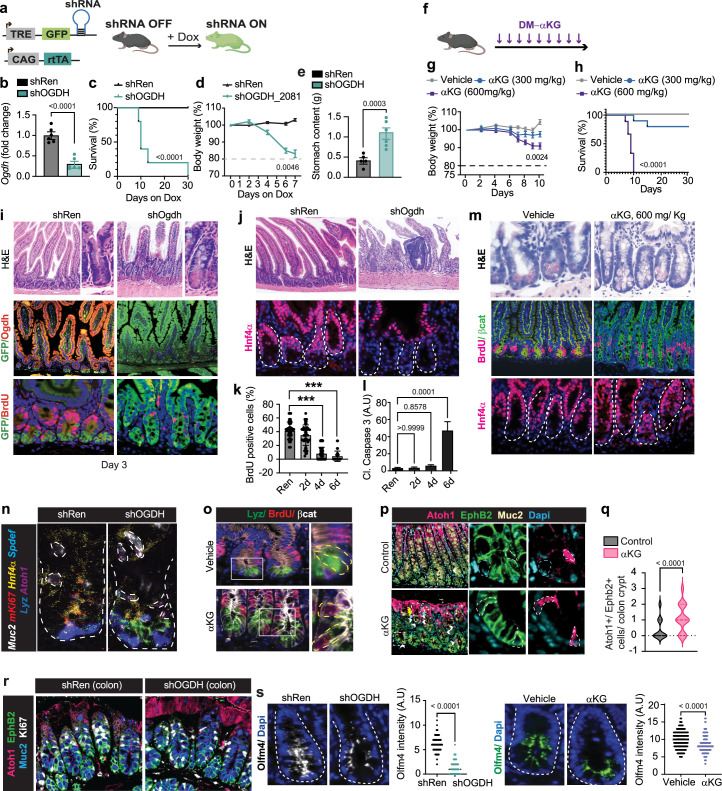
Role of OGDH in intestinal homeostasis in vivo. **a**, Experimental design. OGDH depletion in *TRE*-*shOgdh*^*Cag-rtTA3*^ mice was induced in adulthood. Created in BioRender. Chaves-Perez, A. (2025) https://BioRender.com/1nvuf0r. **b**, qPCR analysis of *Ogdh* expression in *TRE*-*shOgdh*^*Cag-rtTA3*^ and *TRE*-*shRen*^*Cag-rtTA3*^ mice after 3 days on doxycycline. Each dot represents a different mouse (n ≥ 5). **c**, Kaplan–Meier survival curves of *TRE*-*shOgdh*^*Cag-rtTA3*^ (n = 5) and *TRE*-*shRen*^*Cag-rtTA3*^ (n = 6) mice under doxycycline treatment. **d**, Body weight relative to weight at baseline in *TRE*-*shOgdh*^*Cag-rtTA3*^ (n = 5) and *TRE*-*shRen*^*Cag-rtTA3*^ (n = 6) mice. **e**, Weight of food in the stomachs of *TRE*-*shOgdh*^*Cag-rtTA3*^ and *TRE*-*shRen*^*Cag-rtTA3*^ mice after 8 days on doxycycline. Each dot represents a different mouse (n ≥ 5). **f**, Experimental schematic for DM-αKG injections in adult C57Bl/6 mice. Created in BioRender. Chaves-Perez, A. (2025) https://BioRender.com/dimozin. **g**, Body weight relative to weight at baseline in mice treated with vehicle or DM-αKG at indicated doses. **h**, Kaplan–Meier analysis of survival of mice treated with DM-αKG at the indicated doses or with vehicle. **i**, H&E and immunofluorescence for BrdU, GFP and OGDH after 3 days on doxycycline. **j**, H&E and immunofluorescence for HNF4α on day 6 of doxycycline treatment. Dashed lines outline crypt structures. **k**,**l**, Quantification of BrdU (**k**) and Cl. Casp3 (**l**) levels in intestinal sections during doxycycline treatment of *TRE*-*shRen*^*Cag-rtTA3*^ and *TRE*-*shOgdh*^*Cag-rtTA3*^ mice. Each dot represents a crypt (n = 5 mice). **m**, H&E and immunofluorescence for β-catenin (Bcat), BrdU and HNF4α in mice that received 7 days of injections of DM-αKG (600 mg/kg). Dashed lines outline crypt structures. **n**, Representative smFISH image visualizing RNAs of various differentiation markers in intestinal crypts from *TRE*-*shOgdh*^*Cag-rtTA3*^ and *TRE*-*shRen*^*Cag-rtTA3*^. Dashed lines outline crypt structures and specific lineages in the intestinal epithelium. **o**, Immunofluorescence for BrdU, β-catenin and lysozyme (Lyz) after a 2-hour BrdU pulse in mice treated with DM-αKG-treated or vehicle. Dashed lines outline specific lineages within the crypts. **p**, Hyperplex immunofluorescence in colonic sections control and αKG-treated mice in steady-state conditions, showing ATOH1 (marking secretory cells), EphB2 (stem cells) and MUC2 (mature secretory cells). **q**, Quantification of ATOH1^+^/EphB2^+^ double-positive secretory progenitors from **p**. **r**, ABP staining in *TRE*-*shOgdh*^*Cag-rtTA3*^ and *TRE*-*shRen*^*Cag-rtTA3*^ mice. **s**, Immunofluorescence and quantification of OLFM4 intensity (arbitrary units) in *TRE*-*shOgdh*^*Cag-rtTA3*^ and *TRE*-*shRen*^*Cag-rtTA3*^ mice at day 4 of treatment, and in vehicle- and DM-αKG-treated mice at day 7 of treatment. Each dot represents one crypt from n ≥ 4 mice. Dashed lines outline crypt structures. This figure is adapted from our published patent (WO2024229094A1)^50^. Statistical analysis: Data represent mean ± s.e.m. Statistical significance was determined using a two-tailed t-test in **b**,**e**,**q**,**s**. Survival probabilities were estimated using the Kaplan–Meier method, and statistical differences between the survival curves were assessed using the log-rank (Mantel–Cox) test in **c**,**h**. Two-way ANOVA followed by Tukey’s HSD test was used in **d**,**g**. One-way ANOVA followed by Tukey’s HSD test was used in **k, l**. Asterisks denote statistical significance (*P < 0.05, **P < 0.01, ***P < 0.001). Schematics created with BioRender.com. Source Data

To validate the lineage-specific effects of TCA-cycle use in vivo, we examined the effect of *Ogdh* suppression on intestinal homeostasis in *TRE-shOgdh*^*Cag-rtTA3*^ mice (Extended Data Fig. 7a). We disentangled the direct TCA-cycle effects from those mediated by increased αKG by comparing *Ogdh* knockdown with DM-αKG administration^3^. In contrast to the *TRE-shRen*^*Cag-rtTA3*^ controls, *TRE*-*shOgdh*^*Cag-rtTA3*^ mice exhibited severe weight loss and, developed bowel obstructions and required euthanasia 7–11 days after doxycycline treatment (Extended Data Fig. 7b–e). Although a high dose of DM-αKG (600 mg kg^−1^) was toxic, a lower dose (300 mg kg^−1^) was tolerated without significant weight loss or intestinal structure disruption (Extended Data Fig. 7f–h).

**Fig. 4:**
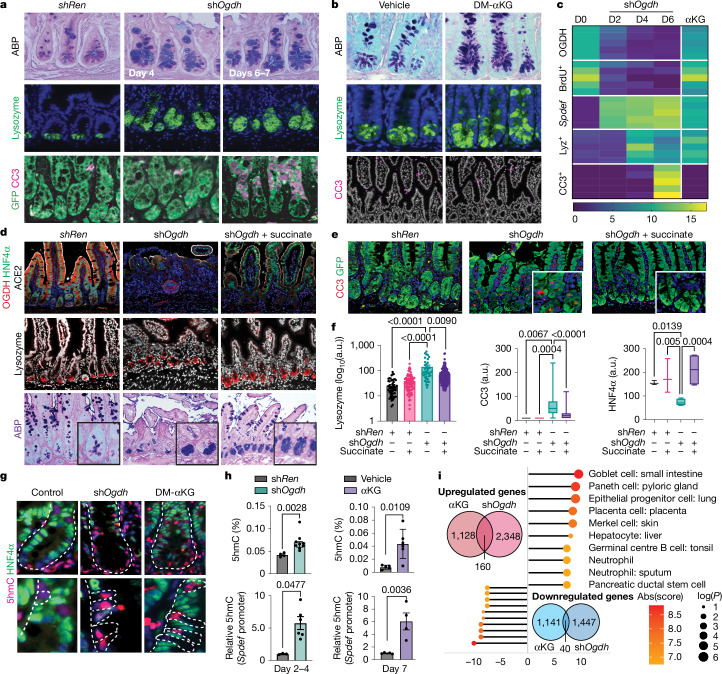
Role of OGDH in gut homeostasis. **a**,**b**, ABP and immunofluorescence for lysozyme, GFP and cleaved caspase 3 (CC3) in intestinal tissue from *TRE*-*shRen*^*Cag-rtTA3*^ and *TRE*-*shOgdh*^*Cag-rtTA3*^ mice (**a**) and vehicle-treated and DM-αKG-treated mice (**b**) at the indicated time points. **c**, Heat map depicting time-course quantification (D indicates day) of OGDH (fluorescence intensity), BrdU (positive cells), *Spdef* (mRNA levels), lysozyme (Lyz) (positive cells) and CC3 (positive cells) in intestinal crypts from doxycycline-treated *TRE*-*shRen*^*Cag-rtTA3*^ and *TRE*-*shOgdh*^*Cag-rtTA3*^ mice or DM-αKG-treated C57Bl/6 mice (*n* = 5 per group). **d**,**e**, ABP and immunofluorescence for OGDH, lysozyme (Paneth cells), HNF4α (enterocytes) and ACE2 (mature enterocytes) (**d**), and CC3 (cell death) (**e**) in intestinal sections from *TRE*-*shOgdh*^*Cag-rtTA3*^ mice concomitantly treated with or without DM-succinate and doxycycline for 6–7 days. Data are representative of sh*Ren n* = 3, sh*Ren* + succinate *n* = 3, sh*Ogdh n* = 8 and sh*Ogdh* + succinate *n* = 4 mice. **f**, Quantification from **e**. Each dot represents one crypt or villus for lysozyme and CC3 and one mouse for HNF4α. a.u., arbitrary units. **g**, Immunofluorescence for 5hmC and HNF4α in crypts from *TRE*-*shRen*^*Cag-rtTA3*^, *TRE*-*shOgdh*^*Cag-rtTA3*^ and DM-αKG-treated mice. Dashed lines outline crypts (top) and 5hmC^+^HNF4α^−^ cells (bottom). **h**, Top, enzyme-linked immunosorbent assay (ELISA) of crypt lysates to measure intestinal 5hmC abundance in *TRE*-*shRen*^*Cag-rtTA3*^, *TRE*-*shOgdh*^*Cag-rtTA3*^, DM-αKG-treated and vehicle-treated mice. Each dot represents one mouse (sh*Ren* *n* = 4, sh*Ogdh* *n* = 9, vehicle *n* = 4, αKG *n* = 6). Bottom, relative 5hmC levels in the *Spdef* promoter within isolated crypts from *shRen*^*Cag-rtTA3*^, *TRE*-*shOgdh*^*Cag-rtTA3*^ and DM-αKG-treated mice, measured by qPCR at the indicated time points. Each dot represents one mouse (*n* ≥ 3 mice per group). **i**, Venn diagrams of upregulated (red) and downregulated (blue) genes in *TRE*-*shOgdh*^*Cag-rtTA3*^ and αKG-treated mice versus controls (*TRE*-*shRen*^*Cag-rtTA3*^ and vehicle-treated), with gene ontology (GO) analysis of the overlapping genes. Abs(score), absolute value of enrichment score. This figure is adapted from our published patent (WO2024229094A1)^50^. Data are mean ± s.e.m. Statistical significance was determined using one-way ANOVA followed by Tukey’s HSD test in **f** and two-tailed *t*-test in **h**. Source Data

Histological and immunofluorescence analysis revealed that αKG supplementation and *Ogdh* knockdown had distinct effects on the physiology of the intestine. *Ogdh* knockdown reduced proliferation within three days of doxycycline treatment (Fig. 4a–c and Extended Data Fig. 7i). Apoptosis occurred later, by day 6, concentrated in the upper region of the crypts and associated with the emergence of crypt hypoplasia and a reduction in HNF4α-positive cells (Fig. 4a–c and Extended Data Fig. 7j–l). Consistent with the high metabolic demand of enterocytes for canonical TCA-cycle functions, supplementation with DM-succinate after OGDH depletion reduced apoptosis (Fig. 4d–f). By marked contrast, treatment with DM-αKG did not induce profound cell-cycle arrest or detectable apoptosis in the crypts (Extended Data Fig. 7m).

**Extended Data Fig. 8:**
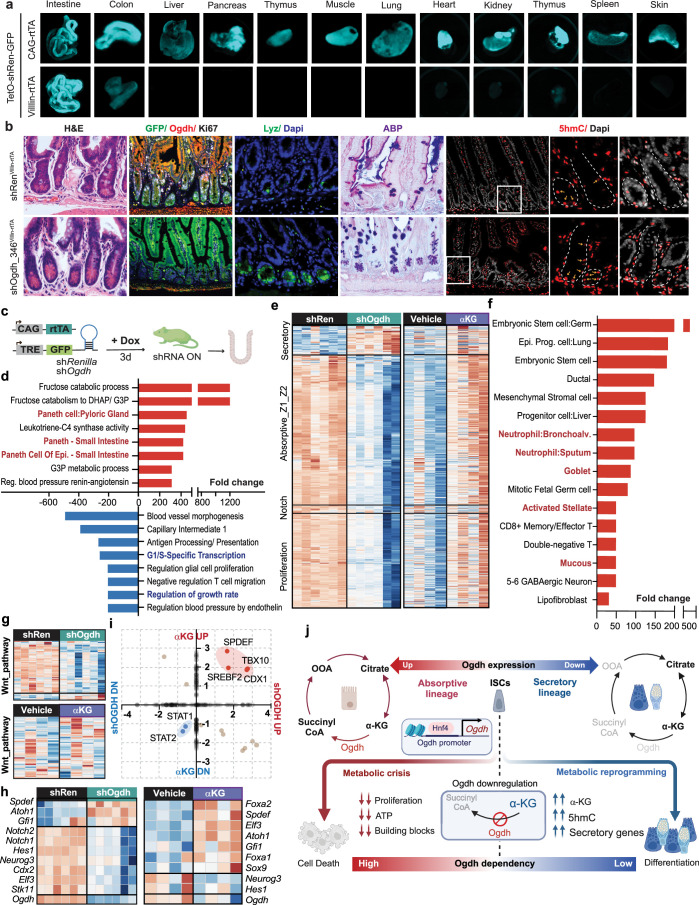
OGDH downregulation increases secretory-lineage specification in vivo*.* **a**, Intestine-specific expression of GFP in *TRE*-*shRen*^*Villin-rtTA3*^ mice. Representative images of GFP fluorescence of various organs from *TRE*-*shRen*^*Cag-rtTA3*^ mice and *TRE*-*shRen*^*Villin-rtTA3*^ mice fed a doxycycline diet for 6 days. **b**, H&E, ABP staining and immunofluorescence of GFP, OGDH, Ki67, lysozyme (Lyz), and 5hmC in intestinal tissues after 5 days of doxycycline treatment in *TRE*-*shOgdh*^*Villin-rtTA3*^ and *TRE*-*shRenilla*^*Villin-rtTA3*^ mice. Dashed lines outline crypt structure. **c**, Design of RNA-seq experiments in isolated intestinal crypts. Created in BioRender. Chaves-Perez, A. (2025) https://BioRender.com/d8uy3en. **d**, Top upregulated and downregulated pathways in *TRE*-*shOgdh*^*Cag-rtTA3*^ versus *TRE*-*shRen*^*Cag-rtTA3*^ mice from GO analysis. **e**, Differential transcription in crypts from *TRE*-*shOgdh*^*Cag-rtTA3*^ and DM-αKG-treated mice compared to *TRE*-*shRen*^*Cag-rtTA3*^ and vehicle-treated mice respectively. The heat map shows genes associated with the secretory lineage, absorptive lineage (zone 1 and zone 2), Notch signalling pathway, and cell proliferation. Each column represents a single mouse. **f**, Top upregulated pathways in intestinal crypts of DM-αKG-treated versus vehicle-treated mice, derived from GO analysis. **g**, Heat map depicting expression of genes in the WNT pathway in crypts from *TRE*-*shOgdh*^*Cag-rtTA3*^, *TRE*-*shRen*^*Cag-rtTA3*^, vehicle-treated and DM-αKG-treated mice after 3 days of treatment. Each lane represents one mouse. **h**, Heat map depicting expression of transcription factors involved in lineage specification in crypts from *TRE*-*shOgdh*^*Cag-rtTA3*^, *TRE*-*shRen*^*Cag-rtTA3*^, vehicle-treated, and DM-αKG-treated mice at day 3 of treatment. **i**, Plot showing predicted transcription factors commonly upregulated (red dots) or downregulated (blue dots) in mice treated with *TRE*-*shOgdh*^*Cag-rtTA3*^ versus DM-αKG. **j**, Schematic depicting the dual role of OGDH in differentiation in intestinal homeostasis. Created in BioRender. Chaves-Perez, A. (2025) https://BioRender.com/7osfp6n. This figure is adapted from our published patent (WO2024229094A1)^50^.

Despite these differences in the absorptive lineage, both αKG supplementation and *Ogdh* suppression triggered an accumulation of 5hmC-high secretory cells and a depletion of ISCs in both the small intestine and the colon (Fig. 4g and Extended Data Fig. 7n–s). Loci-targeted bisulfite sequencing detected increased levels of 5hmC at the *Spdef* promoter, a Paneth cell and goblet cell transcription factor, correlating with early upregulation of *Spdef* (Fig. 4h,i) and a subsequent expansion of secretory cells (Fig. 4a–c and Extended Data Fig. 7n–s). Although the *TRE-shOgdh*^*Cags-rtTA3*^ model induces systemic *Ogdh* suppression, similar effects were observed with intestine-specific *Ogdh* knockdown using *TRE-shOgdh*^*Villin-rtTA*^ mice, which exhibited an increase in lysozyme-expressing secretory cells, along with high levels of ABP and 5hmC (Extended Data Fig. 8a,b).

To further characterize the molecular changes induced by αKG supplementation and *Ogdh* suppression, we performed bulk RNA-seq analysis on isolated intestinal crypts (Extended Data Fig. 8c). *Ogdh* suppression, but not DM-αKG supplementation, significantly reduced transcriptional programs associated with cell proliferation and specification of the absorptive lineage (Extended Data Fig. 8d–i). By contrast, transcriptional signatures of the secretory lineage were enriched in both DM-αKG-treated and *TRE-shOgdh*^*Cag-rtTA3*^ mice (Fig. 4i and Extended Data Fig. 8d–i). These findings indicate that OGDH is essential for enterocyte expansion and survival, and that its suppression contributes to secretory-lineage differentiation. Therefore, regulation of OGDH expression is crucial for lineage specification and balance during intestinal regeneration (Extended Data Fig. 8j).

## Metabolic interventions for tissue repair

**Fig. 5:**
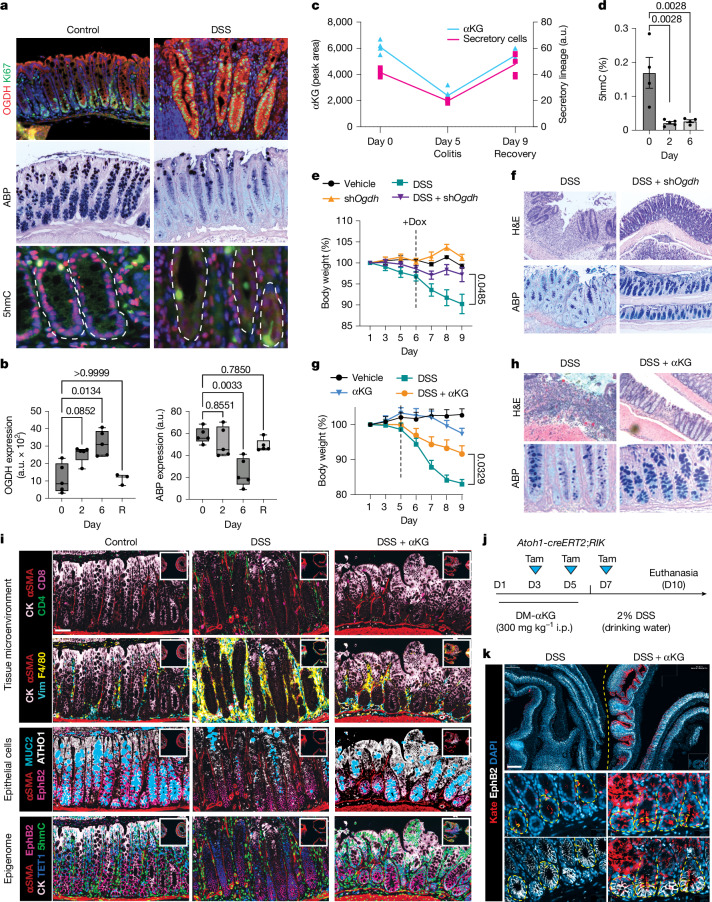
Metabolic interventions to treat ulcerative colitis. **a**, ABP staining and immunofluorescence for OGDH, Ki67 and 5hmC in colon samples from mice with or without colitis induced by DSS. Dashed lines outline colonic crypts. **b**, ABP levels and OGDH expression over time in mice treated with DSS (*n* ≥ 5). The box represents the IQR and the central line represents the median. Whiskers extend to 1.5 × IQR beyond Q1 and Q3. R, recovery (15 days). **c**, Relationship between αKG levels (measured by LC–MS from colonic samples) and secretory cell abundance (measured as number pixels of ABP staining) in DSS-treated mice over time (*n* ≥ 4 per time point and condition). **d**, ELISA from whole-colon lysates to measure 5hmC abundance in DSS-treated mice. Each dot represents one mouse (*n* ≥ 4). **e**, Body weight (relative to initial body weight) in the indicated conditions (vehicle *n* = 4, sh*Ogdh n* = 5, DSS *n* = 7, DSS + sh*Ogdh* *n* = 11). **f**, Haematoxylin and eosin (H&E) and ABP staining of colonic samples isolated from DSS-treated mice or DSS-treated mice with pulsatile OGDH inhibition at day 9. **g**, Body weight under the specified conditions, relative to body weight at baseline (vehicle *n* = 30, αKG *n* = 15, DSS *n* = 35, DSS + αKG *n* = 15). **h**, H&E and ABP staining of colonic sections from DSS-treated or DSS-and-αKG-treated mice at day 9. **i**, Multiplex immunofluorescence under the specified conditions, revealing the architecture and cell composition of the tissue microenvironment, epithelial compartment and epigenetic landscape in DSS-and-αKG-treated-treated mice. CK, pan-cytokeratin; Vim, vimentin; αSMA, α-smooth muscle actin. Scale bar, 50 μm. **j**, Experimental design to trace secretory progenitors (ATOH1^+^ cells) using an Atoh1-CreERT2 line crossed with an LSL Kate reporter mouse. i.p., intraperitoneal; Tam, tamoxifen. **k**, Immunofluorescence for Kate (progeny of ATOH1^+^ cells) and EphB2 (colonic stem cells) under the specified conditions. The dashed lines indicate the boundary between DSS and DSS + αKG intestines within the same slide. Scale bar, 400 μm. This figure is adapted from our published patent (WO2024229094A1)^50^. Data are mean ± s.e.m. Statistical significance was determined by one-way ANOVA followed by Tukey’s HSD test in **b**,**d** and two-way ANOVA followed by Tukey’s HSD test in **e**,**g**. Source Data

**Extended Data Fig. 9:**
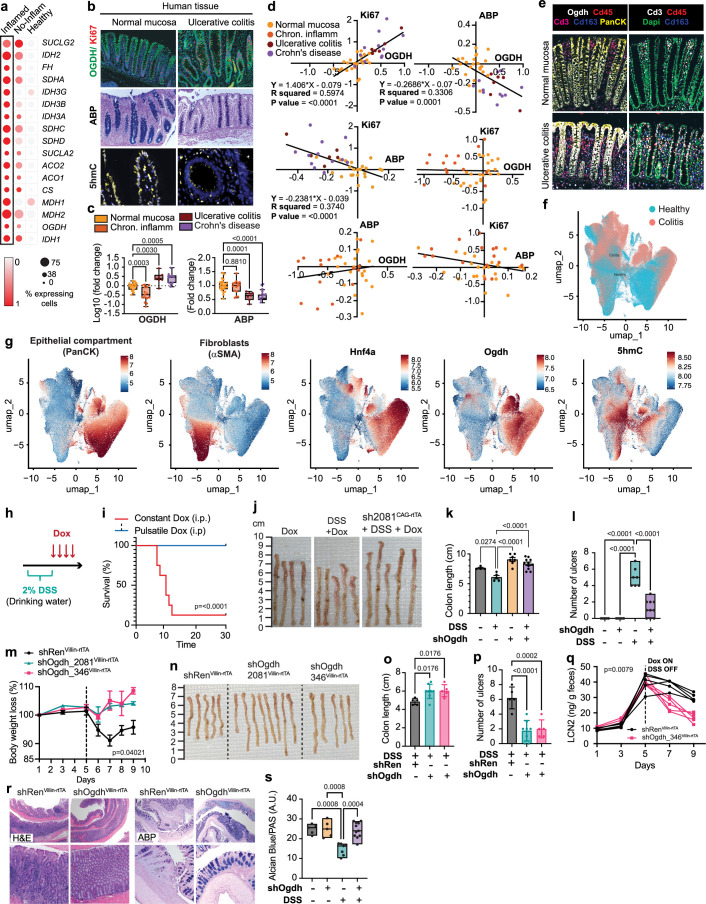
OGDH targeting as an intervention for ulcerative colitis. **a**, Dot plot of RNA expression of TCA-cycle enzymes in human colon from publicly available scRNA-seq data. The plot compares non-inflamed and inflamed tissue from individuals with inflammatory bowel disease and tissue from healthy volunteers. Each column shows expression of the indicated TCA-cycle enzyme. Colour intensity indicates expression level and dot size indicates the proportion of positive cells in that lineage. **b**, Immunofluorescence for OGDH, Ki67 and 5hmC, along with ABP staining in TMAs of human intestinal samples from individuals with or without colitis. **c**,**d,** Quantification (**c**) and correlations of expression (**d**) of OGDH, Ki67 and ABP in normal mucosa, chronic inflammation, ulcerative colitis and Crohn’s disease, based on the TMAs shown in **b**. The top row shows all individuals, and the bottom row shows only those with chronic inflammation or normal mucosa. Correlation coefficients (*r*) were derived from Pearson correlation analysis. The lines represent the best-fit linear regression. The *R*² values indicate the proportion of the variance in disease severity that can be explained by the level of the particular biomarker. Each dot represents a patient. **e**, Representative images from multiplex immunofluorescence analysis of human TMAs, showing the immune compartment (CD45, CD3 and CD163) and the epithelial compartment (OGDH and PanCK) in healthy individuals and patients with ulcerative colitis. **f**,**g**, UMAP illustrating the diversity of cell types in healthy individuals and patients with ulcerative colitis (**f**) and protein distribution (**g**), identified through multiplex immunofluorescence analysis. Each point represents a single cell, colour-coded based on its cell-type classification or expression of the indicated protein. Distinct clusters highlight populations of cells with similar phenotypes. **h**, Experimental scheme of DSS-induced colitis treatment with pulsatile OGDH inhibition in mice. **i**, Kaplan–Meier survival curves for continuous versus pulsatile doxycycline treatment (3 days ON/4 days OFF/week) in *TRE*-*shOgdh*^*Cag-rtTA3*^ mice. **j**, Representative images showing length of colons from *TRE*-*shOgdh*^*Cag-rtTA3*^ mice on day 9 under different conditions. **k**,**l**, Colon length (**k**) and number of ulcers (**l**) at day 9 in the indicated conditions. Each dot represents an independent mouse (n ≥ 4). **m**, Body weight in *TRE*-*shRen*^*Villin-rtTA3*^ (n = 5) and *TRE*-*shOgdh*^*Villin-rtTA3*^ (*n* = 13) conditions relative to weight at baseline. **n**, Representative images showing the length of colons from *TRE*-*shRen*^*Villin-rtTA3*^ and *TRE*-*shOgdh*^*Villin-rtTA3*^ mice after 9 days of the indicated treatments. **o**,**p**, Colon length (**o**) and number of ulcers (**p**) in *TRE*-*shRen*^*Villin-rtTA3*^ and *TRE*-*shOgdh*^*Villin-rtTA3*^ mice at day 9. Each dot represents one mouse (n ≥ 4). **q**, Longitudinal analysis of faecal lipocalin 2 (LCN2) during DSS treatment in *TRE*-*shRen*^*Villin-rtTA3*^ (n = 5*)* and *TRE*-*shOgdh*^*Villin-rtTA3*^ mice (n = 5). Each line represents one mouse. **r**,**s**, H&E and ABP staining (**r**) and ABP quantification (**s**) of intestinal sections from *TRE*-*shRen*^*Villin-rtTA3*^ and *TRE*-*shOgdh*^*Villin-rtTA3*^ mice at day 9 of treatment. This figure is adapted from our published patent (WO2024229094A1)^50^. Statistical analysis: Data represent mean ± s.e.m. Statistical significance was determined using one-way ANOVA followed by Tukey’s HSD test for **c**,**k**,**l**,**o**,**p**,**s**. Survival probabilities were estimated using the Kaplan–Meier method, and statistical differences between the survival curves were assessed using the log-rank (Mantel–Cox) test in **i**. Two-way ANOVA followed by Tukey’s HSD test was used in **m**,**q**. Asterisks denote statistical significance (*P < 0.05, **P < 0.01, ***P < 0.001). Source Data

Perturbed lineage specification during intestinal regeneration contributes to Crohn’s disease and ulcerative colitis, both of which are marked by chronic inflammation and a depletion of mature secretory cells^15,17,41,42^. Given the observed decrease in secretory lineages, we hypothesized that altered OGDH expression might be associated with reduced αKG pools and impaired secretory differentiation and tissue repair. In the dextran sulfate sodium (DSS)-induced model of intestinal injury^43^, mice exhibited increased OGDH expression, enhanced proliferation and decreased secretory-lineage specification (Fig. 5a,b). Furthermore, αKG fell during the injury phase and recovered after tissue healing (Fig. 5c), correlating with reduced levels of 5hmC and fewer secretory cells (Fig. 5c,d). The observations closely mirrored those in human colitis, in which scRNAseq and multiplex IF in human samples revealed increased OGDH-positive cells, reduced secretory cells, increased proliferation and lower levels of 5hmC in inflamed versus normal mucosa^44^ (Extended Data Fig. 9a–g).

Given that increased OGDH was associated with reduced levels of αKG and low numbers of secretory cells in mice with intestinal injury, we hypothesized that epithelial suppression of OGDH could restore αKG pools, enhance secretory differentiation and promote tissue repair. To test this, *TRE-shRen*^*Villin-rtTA3*^ or *TRE-shOgdh*^*Villin-rtTA3*^ mice were treated with DSS for five days, followed by intermittent dosing with doxycycline (four days on and three days off) to preserve enterocyte viability (Extended Data Fig. 9h,i). DSS-treated *TRE-shRen*^*Villin-rtTA3*^ and *TRE-shRen*^*Cag-rtTA3*^ mice exhibited weight loss, colonic shortening, ulceration and increased expression of LCN2, an inflammatory marker associated with colonic damage^17^ (Fig. 5e and Extended Data Fig. 9j–q). By contrast, *Ogdh* suppression in *TRE-shRen*^*Cag-rtTA3*^ and *TRE-shOgdh*^*Villin-rtTA3*^ mice significantly improved colon length, reduced ulceration and LCN2 expression and mitigated weight loss (Fig. 5e and Extended Data Fig. 9j–q). Histological analysis of colons nine days after DSS treatment showed that *Ogdh* suppression enhanced colon structure and increased the abundance of mature goblet cells (Fig. 5f and Extended Data Fig. 9r,s).

**Extended Data Fig. 10:**
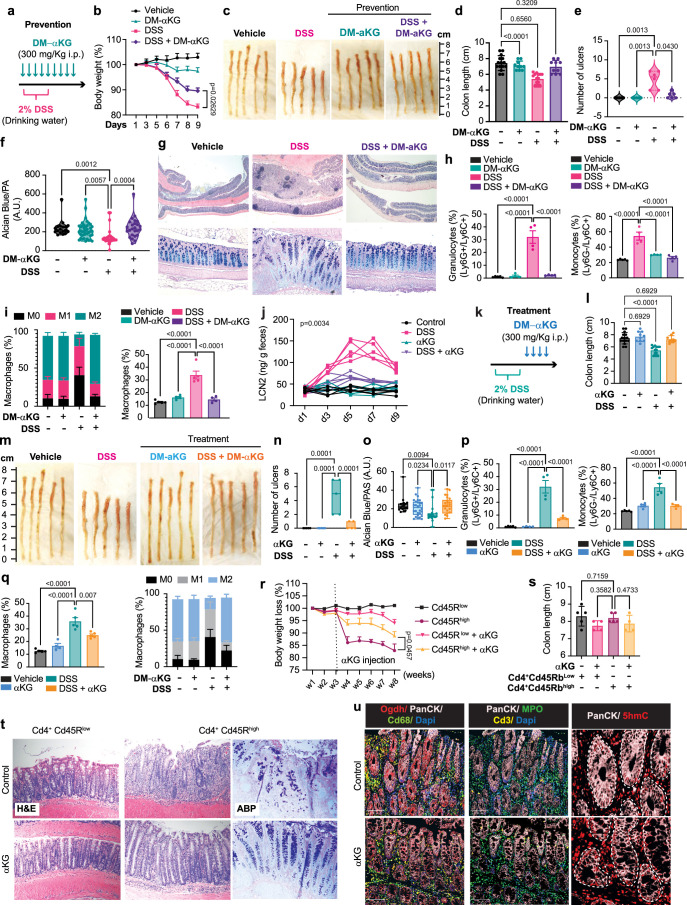
αKG supplementation as an intervention for ulcerative colitis. **a**, Schematic of DM-αKG supplementation as a prevention strategy for ulcerative colitis induced by DSS. **b**, Body weight of mice given the indicated treatments relative to weight at baseline. **c**, Representative images showing length of colons from mice after 9 days of the indicated treatments. **d**, Colon length after 9 days of the indicated treatments. Each dot represents a different mouse (n ≥ 4). **e**,**f**, Number of ulcers (**e**) and ABP quantification (**f**) after 9 days of the indicated treatments. Each dot represents a different mouse (n ≥ 4). **g**, ABP and H&E staining of intestinal sections after 9 days’ treatment of mice with DSS or DSS/αKG. **h**,**i**, Quantification of monocytes and granulocytes (**h**) and macrophages (**i**) by immunophenotyping in αKG prevention settings. Each dot represents a mouse (n ≥ 4). **j**, Longitudinal analysis of faecal LCN2 at indicated time points during DSS treatment in DSS- and DSS/αKG-treated mice. Each line represents one mouse (n ≥ 4). **k**, Experimental plan for DM-αKG supplementation as a treatment for DSS-induced ulcerative colitis. **l**–**o**, Colon length (**l**), representative images of colon length (**m**), numbers of ulcers (**n**) and ABP quantification (**o**) after 9 days of the indicated treatments. Each dot represents a different mouse (n ≥ 4). **p**,**q**, Quantification of monocytes and granulocytes (**p**) and macrophages (**q**) by immunophenotyping in αKG treatment settings. Each dot represents a mouse (n ≥ 4). **r**, Body weight as a percentage of initial weight in the *Rag2*^−/−^ colitis model in the indicated conditions. **s**, Colon length at week 8 in the *Rag2*^−/−^ colitis model under the indicated conditions. Each dot represents a different mouse (n ≥ 4). **t**, ABP and H&E staining of intestinal sections from *Rag2*^−/−^ mice. **u**, Multiplex immunofluorescence of colon sections from *Rag2*^−/−^ CD45Rb-high mice, with or without αKG treatment. Dashed lines outline crypt structures. This figure is adapted from our published patent (WO2024229094A1)^50^. Statistical analysis: Data represent mean ± s.e.m. Statistical significance was determined using one-way ANOVA followed by Tukey’s HSD test for **d**–**f**,**h**,**i**,**l**,**n**–**q**,**s**. Two-way ANOVA followed by Tukey’s HSD test was used in **b**,**j**,**r**. Asterisks denote statistical significance (*P < 0.05, **P < 0.01, ***P < 0.001). Source Data

Consistent with an αKG-driven mechanism, intraperitoneal delivery of 300 mg kg^−1^ DM-αKG restored secretory-lineage specification and improved DSS-induced colitis in both prevention (Extended Data Fig. 10a–j) and intervention (Fig. 5g,h and Extended Data Fig. 10k–q) protocols. Similar results were observed in an immune-mediated colitis model, in which colonic inflammation is produced by the adoptive transfer of CD4^+^ T cells from a healthy donor into *Rag2*^−/−^ mice^45,46^ (Extended Data Fig. 10r–t). Accordingly, multiplexed immunofluorescence on colonic tissue revealed an increase in the secretory lineage after αKG supplementation, and a histologically normal intestinal mucosa in both models (Fig. 5i and Extended Data Fig. 10u). These findings indicate that restoring the levels of αKG in colonic epithelium enhances the differentiation of secretory cells and accelerates tissue repair in colitis.

In addition to protecting against colitis-induced tissue damage, secretory cells can dedifferentiate to replenish the ISC compartment^47^. To investigate whether αKG-induced expansion of secretory progenitor cells (Extended Data Fig. 7n–q) contributes to the enhanced regeneration, we traced the fate of secretory progenitors with and without exogenous αKG during DSS-induced colitis (Fig. 5j). Because *Atoh1* is specifically induced in secretory progenitors and their progeny^47^, we generated double-transgenic mice carrying an *Atoh1* promoter-CreERT2 and a lox-stop-lox (LSL) mKate reporter. After treatment with DSS, tamoxifen-induced labelling revealed that ATOH1^+^ cells could restore the ISC pool (EphB2^+^) and give rise to multilineage reporter-expressing ‘ribbons’ generated from individual secretory progenitors (Fig. 5k). These findings indicate that αKG can influence regenerative plasticity in the intestine beyond its canonical role in the TCA cycle.

## Discussion

Our study highlights the dual role of the TCA cycle in intestinal cell fate, with implications for mucosal healing. Using a powerful mouse model with inducible and reversible *Ogdh* suppression and a flexible organoid culture system, we investigated metabolic adaptations in both ISCs and lineage-specific progenitors, including enterocytes, goblet and Paneth cells^29^. These approaches allowed us to interrogate the function of OGDH in vitro and in vivo and to establish the unique metabolic programs that are required for each lineage. Contrary to the notion that core metabolic enzymes are ‘housekeeping genes’ that are expressed constitutively in all cell types, our study reveals the dynamic and lineage-specific transcriptional regulation of metabolic enzymes. Specifically, we show that the expression of the TCA-cycle enzymes decreases during differentiation into the secretory lineage but increases during differentiation into the absorptive lineage. The increases in OGDH expression are directly controlled by the enterocyte-lineage-defining HNF4 transcription factors, highlighting how transcriptional programs linked to cell fate can rewire metabolism to meet tissue demands.

Perturbation studies reveal a dichotomy in OGDH function across lineages. Consistent with previous work indicating that secretory cells rely less on mitochondrial function^19^, *Ogdh* suppression and subsequent αKG accumulation prime ISCs towards secretory differentiation. By contrast, depletion of OGDH in enterocytes induces cell-cycle arrest and death owing to a disrupted biosynthetic and bioenergetic balance in pAbs. The distinct dependencies of absorptive and secretory lineages on OGDH reflect heterogeneous metabolic demands: absorptive cells probably require higher levels of ATP and TCA-cycle metabolites for effector functions, whereas secretory cells rely on anabolic precursors for producing mucin and antimicrobial peptides. Although our findings support a role for αKG-dependent dioxygenases^2,25^ in secretory differentiation, further studies are needed to clarify how αKG regulates cell fate independently of its TCA-cycle functions. Regardless, these results underscore the role of lineage-specific metabolism in stem cell biology and tissue regeneration, showing that metabolism is both an output and a driver of lineage specification. Similar approaches might be useful for studying other metabolic activities that control lineage-specific cell function and fate.

Our findings also highlight the potential of metabolic interventions to influence cell fate and enhance tissue regeneration during injury. Specifically, we show that increasing αKG levels promotes secretory-lineage differentiation and colonic regeneration, even in the context of chronic inflammation. This suggests that αKG supplementation or partial *OGDH* suppression could restore tissue homeostasis and repair in conditions marked by impaired differentiation^48^ and chronic inflammation. These effects might also explain the reported benefits of αKG in mitigating age-related tissue decline^49^. More broadly, using precise metabolic perturbations to direct cell fate offers a promising avenue for treating enteropathies and advancing tissue rejuvenation strategies.

## Methods

### Mouse models

#### Housing conditions

All animal experiments in this study were performed in accordance with protocols approved by the Memorial Sloan Kettering Institutional Animal Care and Use Committee (approval number: 11-06-012). The mice were housed with a 12-h light–dark cycle between 8:00 and 20:00 in a temperature-controlled room (22 ± 1 °C) with free access to water and food. Both male and female mice were used in equal proportions for all experiments. No sex-based differences were observed. Experiments were performed using mice aged 10–14 weeks. Sample sizes were determined on the basis of previous experiments and published studies to ensure adequate power to detect biologically relevant differences. Mice were randomly assigned to experimental groups. Investigators were blinded to group allocation during data collection and analysis whenever possible.

#### Generation of an inducible *Ogdh*-knockdown mouse

Considering that the *Ogdh* knockout is embryonic lethal^51^, we developed an inducible model using doxycycline-inducible shRNAs linked to a GFP reporter. This system enables temporal and reversible suppression of *Ogdh* expression and facilitates the tracing and analysis of cells with *Ogdh* knockdown using the GFP reporter. To account for potential off-target effects of RNA interference (RNAi), we used two validated shRNAs (sh*Ogdh*_2081 and sh*Ogdh*_346)^1^. As a control for non-sequence-based effects of perturbing the RNAi machinery, we used a similar construct containing a Renilla-luciferase-targeting shRNA (sh*Ren_*731), which does not target any gene expressed in mouse cells. The guide strands for shRNAs are: *Renilla*, TAGATAAGCATTATAATTCCT; *Ogdh*_2081, TAAATGAAACATTTTGTCCTG; *Ogdh*_346, TAGCAATTCTGCATACTTCTG. Doxycycline-inducible GFP-coupled shRNA constructs were electroporated and integrated into a ‘homing cassette’ at the *Col1a1* locus in 4482 ES cells^30,33^; this cassette contains a doxycycline-inducible reverse Tet transactivator (rtTA-M2) expressed from the Cag-rtTA promoter. Validated clones were used to generate chimeric mice using eight-cell aggregation, allowing the assessment of functionality in F_0_. The resulting founders were backcrossed to establish germline transmission, generating Tg. *TRE*-*shOgdh_2081*, Tg. *TRE*-*shOgdh_346* and Tg. *TRE*-*shRenilla_731* mice. To further amplify *Ogdh* knockdown and enable widespread expression of the *TRE*-GFP-shRNA cassette, including in the intestine^33^, we crossed *TRE*-*shRenilla* and *TRE*-*shOgdh* mice with *CAGs-rtTA3* transgenic mice, resulting in *TRE*-*shRenilla*^*Cag-rtTA3*^ and *TRE*-*shOgdh*^*Cag-rtTA3*^ mice.

#### ISC analysis

To enable isolation of LGR5^+^ cells for assessing the expression of TCA-cycle enzymes, we used *Lgr5-EGFP-IRES-creERT2* mice^52^.

#### Generation of a reporter mouse for secretory progenitors

To address whether the secretory pool has plasticity and could undergo dedifferentiation into ISCs, a reporter mouse was generated by crossing the *Atoh1-creERT2* mouse model^47^ with *Rosa26-CAGs-LSL-RIK*^53^, which contains a loxP-flanked stop cassette upstream of the RtTA3, an IRES sequence and the monomeric far-red fluorescent protein mKate2, all inserted into the Gt(ROSA)26 locus. Administration of 4-hydroxytamoxifen leads to the excision of the stop cassette in the RIK allele. Consequently, ATOH1^+^ cells and their progeny will permanently express mKate2, enabling their identification and the dynamic tracking of the fate of ATOH1-expressing cells.

#### Generation of an immune-mediated colitis model (*Rag2*^−/−^ mice)

To generate a rodent model of human inflammatory bowel disease, we used the CD4^+^CD45RB^high^-induced colitis model in *Rag2*^−/−^ mice^45^. In brief, spleens from ten C57Bl/6 male mice were collected, smashed, filtered through a 40-μm filter and washed with isolation buffer (phosphate-buffered saline (PBS), 0.5% bovine serum albumin (BSA) and 2 mM EDTA, pH 7.2). The cells were then centrifuged (288*g*, 5 min), and the resulting splenocyte pellets were resuspended in ACK buffer (Quality Biologicals, 118-156-101CS) to lyse red blood cells. After cell counting, CD4^+^ cells were isolated using the CD4^+^ isolation kit (Miltenyi, 130-104-454) following the manufacturer’s instructions. The splenocytes were transferred to fluorescence-activated cell sorting (FACS) buffer (0.5% BSA and 2 mM EDTA in Ca^2^^+^/Mg^2+^-free PBS) and incubated on ice for 30 min with anti-CD4–APC (BioLegend, 116014, clone RM4-4, 1:200) and anti-CD45Rb–FITC (BioLegend, 103306, clone C363-16A, 1:200) antibodies. CD4^+^CD45Rb^high^ and CD4^+^CD45Rb^low^ cells were then sorted using a Sony MA900 cell sorter. Finally, 0.5 × 10^6^ cells were intraperitoneally injected into *Rag2*^−/−^ male mice, and colitis developed within the following three months.

### Mouse diets and treatments

Cag-rtTA3 mediating sh*Renilla* and sh*Ogdh* expression was activated by feeding mice with a doxycycline hyclate diet (200 mg kg^−1^) at adult stage. The food was changed twice per week.

Acute DSS treatment was performed as previously described^43^. In brief, at ten weeks of age, mice were treated with 2% DSS (MP-Biomedicals, 021160110-CF) in drinking water for five days. Afterwards, the water was changed to regular drinking water and mice were euthanized at the indicated time points. In all experiments with DSS models, mice were weighed at the beginning of DSS treatment and every other day thereafter. In addition, they were evaluated daily for signs of distress or end-point criteria. Specifically, mice were immediately euthanized if they lost more than 20% of their initial body weight or showed breathing difficulties.

For the bromodeoxyuridine (BrdU) pulse experiment, mice were injected with 1 mg of BrdU (Sigma-Aldrich) in PBS and euthanized two hours later.

For DM-αKG supplementation, mice were injected (intraperitoneally) once daily with 600 mg kg^−1^ or 300 mg kg^−1^ of DM-αKG (349631-5G, Sigma-Aldrich) dissolved in PBS. For the vehicle control, mice were injected with PBS.

For pulsatile inhibition of *Ogdh*, mice were injected intraperitoneally with doxycycline (2.5 mg kg^−1^, Sigma-Aldrich, D9891) once daily in cycles of three consecutive days on followed by four days off.

DM-succinate treatment was performed as described^54^. In summary, mice were provided with 100 mM DM-succinate (W239607-1KG-K, Sigma-Aldrich) in their drinking water and received intraperitoneal injections of 100 mM DM-succinate every other day throughout the experiment. The pH was adjusted to 6.5 for both the drinking water and the injections.

For pulse-chase labelling experiments in *Atoh1-creERT2*;*RIK* mice, 5 mg of tamoxifen (T5648-1, Sigma-Aldrich) per mouse was administered by oral gavage every other day for a total of five days.

For glycolysis inhibition, mice were treated with intraperitoneally injected with 500 mg kg^−1^ of 2-deoxyglucose (Sigma, 111980050) five times per week for one month.

### Genotyping PCR

Genomic DNA was extracted from mouse tails and ear punches. Biopsies were digested in MGB buffer supplemented with 10% Triton X-100, 1% 2-mercaptoethanol and 0.4 mg ml^−1^ proteinase K (Qiagen, 19133), and incubated overnight at 55 °C. PCR conditions were 95 °C for 6 min, then 35 cycles (95 °C for 40 s, 62 °C for 45 s, 72 °C for 1 min), then 72 °C for 10 min. PCR amplification yielded a 300-bp band for the mutant allele (indicating successful shRNA integration) and a 218-bp band for the wild-type allele.

### RNA-seq analysis and qPCR

#### RNA extraction and RNA-seq library preparation and sequencing

Total RNA was isolated from crypts isolated from *TRE-shRenilla*^*Cag-rtTA3*^, *TRE-shOgdh*^*Cag-rtTA3*^, vehicle-treated or DM-αKG-treated mice using RNeasy kits (QIAGEN, 74004). RNA concentration and quality was assessed using an Agilent 2100 Bioanalyzer. Sequencing and library preparation was performed at the Integrated Genomics Operation at the Memorial Sloan Kettering Cancer Center (MSKCC). RNA-seq libraries were prepared from total RNA. After RiboGreen quantification and quality control by Agilent Bioanalyzer, 100–500 ng of total RNA underwent polyA selection and TruSeq library preparation according to the instructions provided by Illumina (TruSeq Stranded mRNA LT Kit, RS-122–2102), with eight cycles of PCR. Samples were barcoded and run on a HiSeq 4000 or HiSeq 2500 in a 50-bp/50-bp paired-end run, using the HiSeq 3000/4000 SBS Kit or TruSeq SBS Kit v4 (Illumina) at MSKCC’s Integrated Genomics Operation. An average of 41 million paired-end reads was generated per sample. Ribosomal reads represented at most 0.01% of the total reads generated, and the fraction of mRNA averaged 53%.

#### RNA-seq read mapping, differential expression analysis and heat-map visualization

Adaptor sequences were removed from the RNA-seq data using Trimmomatic^55^. The trimmed reads were then aligned to the GRCm38.91 (mm10) reference genome using STAR^56^, and the transcript count was quantified using featureCounts^57^ to generate a raw count matrix. Differential gene expression analysis was performed using the DESeq2 package^58^ in R (http://cran.r-project.org/), comparing the experimental conditions. Each condition had three to five independent biological replicates (individual mice). PCA was performed using DESeq2 to visualize the variation in gene expression among the samples. Differentially expressed genes (DEGs) were identified on the basis of a greater than twofold change in gene expression with *P*_adj_ < 0.05. To visualize the DEGs, the samples were *z*-score normalized and plotted as a heat map using the ‘pheatmap’ package in R. Functional annotations of the gene sets were done by pathway enrichment analysis using the Reactome, Azimut, CellType and KEGG databases. The analysis was performed with enrichR^59^, and the significance of the tests was assessed using a combined score, calculated as log(*P*) × *z*, where *P* represents the *P* value from the Fisher exact test, and *z* is the *z*-score indicating the deviation from the expected rank. Gene set enrichment analysis (GSEA)^60^ was done using the GSEA-Preranked tool (v.2.07). The analysis involved the enrichment of gene sets using RNA-seq data obtained from the experiment. The gene sets were derived from the MSigDB database (http://software.broadinstitute.org/gsea/msigdb), as well as previously published signatures from mouse intestine.

### scRNA-seq data analysis

Data were obtained from a previously published study^26–28^. In brief, human intestines were collected from three male donors (aged 29, 45 and 53 years) through HonorBridge (formerly Carolina Donor Services). Donors met eligibility criteria, including the absence of infectious diseases, cancer or recent abdominal surgeries. Intestinal tissues were divided into six regions: duodenum, jejunum, ileum and the ascending, transverse and descending colon. Mucosectomies (3 × 3 cm) were taken from the centre of each region. Sample preparation and cell hashing were performed as described using the reported cell classification and anatomical location^26–28^. Raw and normalized data, along with cell annotations, were obtained from a published study and downloaded from GSE185224. Seurat^60–63^ was used to perform the scRNA-seq data analysis, and the.h5ad file was converted into a Seurat object using the R package zellkonverter. The GetAssayData function was used to extract *Ogdh* expression, and the AverageExpression function was used to calculate the average expression across different regions or lineages. The TCA-cycle gene set was generated using the genes from the TCA-cycle enzymes, and the gene signature score was computed using the AddModuleScore function from Seurat. Gene signatures used in the paper are shown in Supplementary Table 8.

### qPCR with reverse transcription

For qPCR with reverse transcription (qRT–PCR) analysis, total RNA was extracted from mouse ES cells, isolated crypts or sorted cells from *Lgr5-EGFP* mice using the RNeasy Mini Kit (QIAGEN, 74004). Subsequently, cDNA synthesis was performed using TaqMan reverse transcription reagents (Applied Biosystems). qPCR was performed in triplicate using SYBR Green PCR Master Mix (Applied Biosystems) on the ViiA 7 Real-Time PCR System (Invitrogen). The expression levels of target genes were normalized to endogenous control genes, *Rplp0* (also known as 36B4) and *Actb*. Gene-specific primer sets were designed using the qPrimerDepot tool provided by the National Center for Biotechnology Information (NCBI) (https://www.ncbi.nlm.nih.gov/tools/primer-blast/) or published elsewhere. For primer sequences and details, see Supplementary Table 2.

### Bisulfite conversion, detection of 5mC and 5hmC loci, primer design and normalization

To quantify 5mC and 5hmC at SPDEF-specific loci, we used the BisulPlus Loci 5mC & 5hmC Detection PCR Kit (P-1067-48), following the manufacturer’s protocol. For DNA extraction and bisulfite conversion:, genomic DNA was extracted from intestinal crypts using the Zymo Research Quick-DNA Miniprep Plus Kit (D4069) and quantified with a NanoDrop spectrophotometer (Thermo Fisher Scientific). For each reaction, 500 ng genomic DNA was subjected to bisulfite conversion to deaminate cytosines while preserving 5mC and 5hmC. The conversion was performed according to the manufacturer’s guidelines. For primer design, primers for bisulfite-converted DNA were designed using MethPrimer (https://methprimer.com/cgi-bin/methprimer/methprimer.cgi/) (Supplementary Table 7). The primer design avoided CpG dinucleotides to minimize bias between methylated and unmethylated DNA. Primers were 26–35 bases long to ensure specificity, with melting temperatures (*T*_m_) adjusted to higher than 60 °C through guanine-rich sequences. Each primer set amplified one strand of the bisulfite-converted DNA. For PCR amplification and detection of 5mC and 5hmC, after bisulfite treatment, the targeted loci were amplified in a 50-µl PCR reaction containing 25 µl of 2× Master Mix, 2 µl of bisulfite-converted DNA, 1 µl of each primer (10 µM) and nuclease-free water. The thermal cycling conditions were: initial denaturation: 95 °C for 5 min; 35 cycles of 95 °C for 30 s (annealing temperature specific to primers) and 72 °C for 1 min; final extension: 72 °C for 5 min. For normalization of 5hmC levels: to accurately quantify 5hmC, PCR signals from the 5hmC-specific reactions were normalized against the total methylation (5mC + 5hmC) at each locus. The percentage of 5hmC was calculated using the following formula: 5hmC (%) = 100 × (5hmC signal/(5mC signal + 5hmC signal)).

For qPCR, the *C*_t_ values of the 5mC- and 5hmC-specific reactions were compared, and the relative levels of 5hmC were obtained using the Δ*C*_t_ method. The difference between *C*_t_ values (Δ*C*_t_) provided an estimate of relative hydroxymethylation, and the fold change was calculated using the formula 2^−ΔCt2^.

### Genetic constructs and plasmids

For plasmid maps and shRNA sequences, see Supplementary Table 6.

The lentiviral vector used to silence *Hnf4a* and *Hnf4g*, pLV[2miR30]-Hygro-TRE3G>mCherry:{shHNF4a}:{shHNF4g}, and its control vector, pLV[2miR30]-Hygro-TRE3G>mCherry: Scramble [miR30-shRNA#1]:Scramble [miR30-shRNA#2], were constructed and packaged by VectorBuilder. These are tandem shRNA expression vectors, each carrying two shRNA sequences designed to be co-expressed under the same regulatory elements, enabling simultaneous knockdown of two target genes or, in the case of the control, two non-targeting scramble sequences. The vector IDs are VB231003-1530dwr (shControl vector) and VB231003-1530srj (*Hnf4a* and *Hnf4g* vector), respectively. These IDs can be used to retrieve specific information about the plasmids. sh*Hnf4a*: TACTATTTTACCTACCTATGGG; sh*Hnf4g*: TTAATATTTATGTCAGTGCTGG; sh*Renilla*: ACCTAAGGTTAAGTCGCCCTCG (×2 tandem sequence).

The reporter vectors used to study the role of the HNF4 family in *Ogdh* expression, pRP[Pro]-hRluc/Puro-{Ogdh_Promoter_WT}>TurboGFP and pRP[Pro]-hRluc/Puro-{Ogdh_Promoter_Hnf4a_Mutant}>TurboGFP, were constructed by VectorBuilder. The vector IDs are VB231003-1479yab and VB231003-1483kbd, respectively. Wild-type *Ogdh* promoter: AACAAGTGTTCAAAATAGTCACTCATGTTATTCAAATTATTTTGTGCAGGGATACATTTTACCAACCCAACTATTATTTAGGGCAGCTTGTTTTGGATACAAAGCCCGTGGGCCTCAAAGTCGCAGCGCTCCTGCTTCGGCCCGCCAAACGCTTCAATTATCAGACGGCATCCCACGCCCTGAATGTACCAGGTTCTTAACAAGCTTCGGAAGCGTCTCCCGTGTAACTGCTAATGACAGCCGAAAGACAGTGAGCAACAGGCTGGCTTTGGCCAGATGCAAAGTTCTGCATTGGCGCGAAGCCCGAGCGAGCGACTGAAACCCAATTCTGTGACGTCACGTCACGCCCACAGCCTGTCTTGCAGGCCGCTCCTCTGGGGCCGGGCTACGCGTTGACGCC. Mutated *Ogdh* promoter: AACAAGTGTTCAAAATAGTCACTCATGTTATTCAAATTATTTTGTGCAGGGATACATTTTACCAACCCAACTATTATTTAGGGCAGCTTGTTTTGGATACAAAGCCCGTGGGCCTCAAAGTCGCAGCGCTCCTGCTTCGGCCCGCCAAACGCTTCAATTATCAGACGGCATCCCACGCCCTGAATGTACCAGGTTCTTAACAAGCTTCGGAAGCGTCTCCCGTGTAACTGCTAATGACAGCCGAAAGACAGTGAGCAACAGGCTGGGGGAGCTAGATGGGGCGATTTGCATTGGCGCGAAGCCCGAGCGAGCGACTGAAACCCAATTCTGTGACGTCACGTCACGCCCACAGCCTGTCTTGCAGGCCGCTCCTCTGGGGCCGGGCTACGCGTTGACGCC.

### Intestine preparation

#### For immunofluorescence analysis

Small intestine and colon were removed from mice and flushed with PBS. Intestines were then opened longitudinally, ‘Swiss-rolled’, incubated overnight in 10% formalin at room temperature, changed to 70% ethanol and processed for paraffin embedding.

#### For isolation of crypts

From freshly dissected small intestines from C57Bl/6, *TRE-shOgdh*^*Cag-rtTA3*^ and *TRE-shRen*^*Cag-rtTA3*^ mice, the duodenum (first 10–12 cm from the stomach) was isolated, as it yields more crypts in a shorter time. The duodenum was longitudinally opened through the lumen, then washed in ice-cold Hanks’ balanced salt solution (HBSS) until the rest of the samples were collected. Next, the duodenum was transferred to a 15-ml Falcon tube, then chopped into pieces of 1–2 cm. For each tube, 6–7 ml of ice-cold 8 mM EDTA in HBSS was added, and the tube was incubated on ice for 15 min. After incubation, the EDTA solution was removed and replaced with HBSS. The tube was then vigorously shaken for approximately 20 s. A 20-μl aliquot was taken from the supernatant and examined under a microscope to confirm that it contained the villus fraction. The supernatant was discarded, and the intestine pieces were then transferred to a new 15-ml Falcon tube and rinsed in ice-cold HBSS to remove any residual villi. Another 6–7 ml of ice-cold 8 mM EDTA in HBSS was added per tube, followed by a 15-min incubation on ice. As in the previous step, the EDTA solution was removed and replaced with HBSS (optional step), and the tube was shaken vigorously for approximately 20 s. A 20-μl aliquot of the supernatant was taken and examined under the microscope to check whether it contained the desired crypt fraction. Depending on the yield, these steps were repeated until the crypts were enriched in the supernatant.

### Culture of intestinal organoids

#### Regular conditions

Freshly isolated crypts were embedded in 50 μl of undiluted Matrigel (356231, Cultek) and cultured in DMEM/F-12 (D8437, Sigma-Aldrich) supplemented with penicillin–streptomycin, 1× (2 mM) Glutamax (35050038, Gibco or Life Technologies, Thermo Fisher Scientific), 10 mM HEPES (15630049, Gibco or Life Technologies, Thermo Fisher Scientific), 2 mM *N*-acetyl cysteine (A8199-10G, Sigma-Aldrich), 1× B27 supplement (17504-044, Life Technologies), 10 mM nicotinamide (N0636-100G, Sigma-Aldrich), 50 ng ml^−1^ recombinant mEGF (PMG8044, Gibco), 100 ng ml^−1^ recombinant Noggin (250-38, PeproTech), 1 μg ml^−1^ mouse R-spondin 1 (120-38, PeproTech) and 1% normocin (ant-nr-1, InvivoGen)^64^. During the initial 12-h culture establishment, 1.5 μM CHIR99021 (GSK3 inhibitor, 2520691; PeproTech) and 10 μM Y-27632 inhibitor (1293823, PeproTech) were included, and then the medium was replaced. The medium was changed every other day.

#### Organoid differentiation

Organoid differentiation assays were performed as previously described^29^. In brief, crypts or single cells were entrapped in Matrigel and plated at the centre of wells in a 24-well plate. After polymerization of Matrigel, 500 μl of complete Advanced DMEM/F-12 was added. The enrichment media were as follows. ISC enrichment medium (ENR-CV): EGF (50 ng ml^−1^, Life Technologies), Noggin (100 ng ml^−1^, PeproTech), R-spondin 1 (500 ng ml^−1^, R&D) and small molecules including CHIR99021 (3 μM, Stemgent) and valproic acid (1 mM, Sigma-Aldrich). Paneth cell enrichment medium (ENR-CD): EGF (50 ng ml^−1^, Life Technologies), Noggin (100 ng ml^−1^, PeproTech), R-spondin 1 (500 ng ml^−1^, R&D) and small molecules including CHIR99021 (3 μM, Stemgent) and DAPT (10 µM, Sigma-Aldrich). Goblet cell enrichment medium (ENR-VD): EGF (50 ng ml^−1^, Life Technologies), Noggin (100 ng ml^−1^, PeproTech), R-spondin 1 (500 ng ml^−1^, R&D) and small molecules including valproic acid (1 mM, Sigma-Aldrich) and DAPT (10 µM, Sigma-Aldrich). Enterocyte enrichment medium (ENR-IV): EGF (50 ng ml^−1^, Life Technologies), Noggin (100 ng ml^−1^, PeproTech), R-spondin 1 (500 ng ml^−1^, R&D) and small molecules including valproic acid (1 mM, Sigma-Aldrich) and IWP2 (2 μM, Sigma-Aldrich). To enrich in progenitor cells, organoids were kept in ENR-CV for six days and then transferred for three days to the corresponding differentiation medium. For fully mature lineages, ISC-enriched organoids were maintained in differentiation medium for at least six days.

### Intestinal organoid engineering

#### Organoid nucleofection protocol

Freshly intestinal organoids were isolated as previously described. They were maintained in ENR medium with CHIR99021 (10 μM) for four days before electroporation. Five drops of Matrigel (30–40 μl) were plated per six-well plate, and the medium was refreshed the day before electroporation. On the day of electroporation, organoids were recovered from Matrigel using Cell Recovery Solution (Corning, 76332-050) and spun down at 135*g* for 5 min at 4 °C. The organoids were then resuspended in 200 μl TrypLE (Life Technologies, 12604021) per well and incubated for 3 min at 37 °C in a water bath. Subsequently, 5 ml PBS was added, and the organoids were further mechanically dissociated. For nucleofection, we used 2–3 μg of the desired plasmid; specifically, a reporter construct carrying the HNF4-binding site in the *Ogdh* promoter, either mutated or wild type. The P3 Primary Cell 4D-Nucleofector X Kit (Lonza, V4XP-3024) was used for nucleofection. The nucleofection buffer was prepared immediately before the procedure, following the manufacturer’s instructions. Organoids were resuspended in 20 μl nucleofection buffer and immediately subjected to electroporation using the ESC program in the 4D-Nucleofector (Lonza). After electroporation, 100 μl ENR medium with CHIR99021 and Y-27632 inhibitors was added to each well. The nucleofected organoids were transferred to an Eppendorf tube, spun down at 1,200 rpm for 5 min and resuspended in Matrigel. Organoids were allowed to recover overnight before experiments.

#### Organoid viral transduction protocol

Organoids were isolated as described above and maintained in ENR medium supplemented with CHIR99021 (10 μM) for four to six days. Next, double *Hnf4a* and *Hnf4g* shRNA along with their respective controls, were transduced by spinoculation, which was performed as previously described^64^. After spinoculation, organoids were spun down at 1,200 rpm for 5 min and resuspended in Matrigel. The organoids were then allowed to recover before selection or sorting.

### Organoid treatments

For the experiments using organoids, the following treatments were applied: doxycycline at 2 μg ml^−1^ (Sigma-Aldrich, D9891-25G), DM-αKG at 3.35 mM (1:2,000 dilution from stock) (Sigma-Aldrich, 102418940), octyl-αKG at 10 mM final concentration (Sigma-Aldrich, 876150-14-0), octyl-L-2HG at 10 mM final concentration (Sigma-Aldrich, 1391194-64-1) and DM-succinate at 3 mM final concentration (Sigma-Aldrich, W239607).

### Metabolomics

#### Intestinal organoid culture

Intestinal organoid culture was performed by isolating crypts through mechanical disruption with EDTA, followed by embedding them in Matrigel. To ensure consistent numbers of cells, crypts from five independent mice were pooled together, and the resulting pool was embedded in Matrigel. Five Matrigel drops (30–40 µl per drop) were plated in every six-well plate. Triplicate samples were plated from the pooled crypts to maintain consistency in the experiments, because the LC–MS is highly sensitive to variations in cell number, and it is challenging to plate the same number of organoids from different mice. Organoids were kept in the pertinent culture medium as described above. Also, as an internal control for the metabolites present in Matrigel, in LC–MS experiments, we isolated metabolites from Matrigel (same number of drops) without organoids but going through the same culture protocol. For isotopologue tracing, organoids were transferred to ENR-X medium containing either ^13^C_6_ glucose or ^13^C_5_ glutamine, without Glutamax, and incubated for 24 h before sample collection.

#### Sample preparation

Supernatant was aspirated from Matrigel cultures with or without organoids. The cells were lysed and metabolites were extracted by adding 1 ml ice-cold 80% methanol directly to the Matrigel drops, followed by incubation overnight at −80 °C to aid protein precipitation. The following day, the methanol extracts were centrifuged at 20,000*g* for 20 min at 4 °C, and 800 µl of the supernatant was transferred to a new tube and evaporated in a vacuum concentrator (GeneVac).

#### LC–MS/MS analysis

For metabolomic profiling using LC–MS, dried extracts were resuspended in 80 µl of 40% acetonitrile in water + 20 µl of 100% methanol for hydrophilic interaction liquid chromatography (HILIC) or in 100 µl of 50% methanol in water for ion-pair LC separations. Samples were vortexed and incubated on ice for 20 min, vortexing every 5 min to ensure adequate resuspension. All samples underwent one final centrifugation (20,000*g* for 20 min at 4 °C) to remove any residual particulate.

HILIC LC–MS analysis was performed on a 6545 Q-TOF mass spectrometer (Agilent Technologies) in positive ionization mode. LC separation was done on an ACQUITY UPLC BEH Amide column (150 mm × 2.1 mm, particle size 1.7 μm, Waters) using a gradient of solvent A (10 mM ammonium acetate in 10:90 acetonitrile: water with 0.2% acetic acid, pH 4) and solvent B (10 mM ammonium acetate in 90:10 acetonitrile: water with 0.2% acetic acid, pH 4). The gradient was 0 min, 95% B; 9 min, 70% B; 13 min, 30% B; 14 min, 30% B; 14.5 min, 95% B; 15 min, 95% B; and 20 min, 95% B. Other LC parameters were as follows: flow rate 400 μl min^−1^, column temperature 40 °C and injection volume 5 μl. Other MS parameters were as follows: gas temperature 300 °C, gas flow 10 l min^−1^, nebulizer pressure 35 psi, sheath gas temperature 350 °C, sheath gas flow 12 l min^−1^, Vcap 4,000 V and fragmentor 125 V.

Ion-pair LC–MS analysis was performed on a 6230 TOF mass spectrometer (Agilent Technologies) in negative ionization mode. LC separation was done on an XSelect HSS T3 column (150 mm × 2.1 mm, particle size 3.5 μm, Waters) using a gradient of solvent A (5 mM octylamine in water with 5 mM acetic acid) and solvent B (5 mM octylamine in methanol with 5 mM acetic acid), and post-column solvent with 90:10 acetone: DMSO. The gradient was at 0.3 ml min^−1^: 0 min, 1% B; 3.5 min, 1% B; 4 min, 35% B; 15 min, 35% B; 20 min, 100% B; at 0.4 ml min^−1^: 20.1 min, 100% B; and 22 min, 100% B, 22.1 min, 1% B; and 27 min, 1% B. Other LC parameters were as follows: post-column flow rate 0.3 ml min^−1^, column temperature 40 °C and injection volume 5 μl. Other MS parameters were as follows: gas temperature 250 °C, gas flow 9 l min^−1^, nebulizer pressure 35 psi, sheath gas temperature 250 °C, sheath gas flow 12 l min^−1^, Vcap 3,500 V and fragmentor 125 V.

Targeted data analysis was performed using both Skyline and MassHunter Profinder software v.10.0 (Agilent Technologies). Further analysis was done using MetaboAnalyst (https://www.metaboanalyst.ca/MetaboAnalyst/ModuleView.xhtml).

#### D-2HG and L-2HG extraction and analysis

Metabolites were extracted with ice-cold 40:40:20 acetonitrile:methanol:water containing 5 μM L- or D-2-hydroxyglutaric acid-d3 disodium salt (Toronto Research Chemicals, H942578) as an internal standard. After overnight incubation at −80 °C, organoid extract was collected, sonicated and centrifuged at 20,000*g* for 20 min at 4 °C to precipitate protein. Extracts were then dried in an evaporator (Genevac EZ-2 Elite. For LC–MS analysis, dried samples were derivatized with 100 μl freshly prepared 50 mg ml^−1^ (+)-diacetyl-l-tartaric anhydride (DATAN; Sigma) in dichloromethane acetic acid (v/v = 4:1) at 75 °C for 30 min. After cooling to room temperature, derivatized samples were dried under nitrogen at room temperature and resuspended in 100 μl UltraPure water (18.2 MΩ, PureLab) before LC–MS/MS analysis. LC–MS analysis was performed on a Thermo Vantage triple-quadrupole mass spectrometer operating in selected reaction monitoring and negative ionization modes using an Acquity UPLC HSS T3 analytical column (2.1 × 100 mm, 1.8 μm, Waters) with an Agilent 1260 infinity binary pump, and applying a gradient of mobile phase A (125 mg l^−1^ ammonium formate in water adjusted to pH 3.5 with formic acid) and mobile phase B (100% methanol) at a flow rate of 300 μl min^−1^. The analytical gradient was 0–5 min, 3% B; 5.5–8 min, 80% B. The column was then re-equilibrated for 10 min to ensure retention-time stability. Other LC parameters were as follows: flow rate 300 μl min^−1^, column temperature 40 °C, sample storage temperature 4 °C and injection volume 10 μl. MS source parameters were as follows: spray voltage 2,500 V, capillary temperature 300 °C, vaporizer temperature 250 °C, sheath gas pressure 50 psi and auxiliary gas pressure 40 psi. Compound-specific S-lens values were as follows: 37 V (2HG) and 41 V (deuterated 2HG). Individual reactions were monitored, and collision energies (CEs) were as follows: 2HG *m*/*z* 363.0–147.1 (CE: 12 V), 129.1 (CE: 27 V); deuterated 2HG *m*/*z* 368.0–152.1 (CE: 13 V), 132.9 (CE: 22 V). The identities of metabolite enantiomers were determined by comparing with the retention times of the derivatized pure standards. Chromatograms were acquired and processed with TraceFinder software (Thermo Fisher Scientific).

### Mitochondria stress test and substrate oxidation assay (Seahorse assays) in organoids

#### Crypt extraction and organoid preparation

Crypts were extracted as described above. Freshly isolated crypts were resuspended in 1.5 ml Matrigel and kept on ice to prevent the Matrigel from polymerizing during the plating process. The number of organoids to plate was first optimized by testing 1 μl, 2 μl or 3 μl of Matrigel with organoids, and thereafter 2 µl was plated. Samples were plated in the centre of a 96-well Seahorse plate, positioned between the three dots. To ensure consistent conditions, in control wells, the same volumes of Matrigel were plated. To account for variations in plating the same number of organoids in each well, an entire column per condition was plated to have eight replicates. Appropriate organoid culture medium was added to each well, and the plate was kept in the incubator until the day of the experiment. Typically, the organoids were allowed to adapt for two days before the experiment was performed.

#### Seahorse experiment

The assay was performed as previously described with some adaptations^65,66^. In brief, the day before the experiment, we prepared complete Seahorse medium: Agilent Seahorse XF (pH 7.4) without phenol red and supplemented with glucose (10 mM), pyruvate (1 mM) and glutamine (2 mM). All reagents were adjusted to a pH of 7.4. The sensor plate was activated in water overnight at 37 °C. On the day of the experiment, the organoids were prepared by removing the organoid complete medium and washing twice with complete Seahorse medium. The complete Seahorse medium was added to the organoids, and they were allowed to adapt for 30 min (maximum one hour) at 37 °C in a non-CO_2_ incubator.

#### Injections and settings

For both MitoStress assays (103015-100) and substrate oxidation assays, the following concentrations were injected into the ports: oligomycin (port A) at 50 μM, FCCP at 20 μM and rotenone and antimycin A at 20 μM each. The settings of the XF Analyzer for the assay were as follows: basal (three cycles) with a mix time of 4 min, a wait time of 0 min and a measure time of 3 min; oligomycin (six cycles) with a mix time of 4 min, a wait time of 0 min and a measure time of 3 min; FCCP (three cycles) with a mix time of 4 min, a wait time of 0 min and a measure time of 3 min; and rotenone and antimycin A (three cycles) with a mix time of 4 min, a wait time of 0 min and a measure time of 3 min. For the long-chain fatty acid oxidation stress test (103672-100), we used etomoxir at a concentration of 20 μM supplemented with 500 μM of carnitine; for the glucose/pyruvate oxidation stress test (103673-100) we used UK5099 inhibitor at 4 µM; and for the glutamine oxidation stress test (103674-100) we used CB839 inhibitor at 3 µM.

#### Normalization

A 4× bright-field picture was taken from all wells to check organoid positioning after the assay. For normalization, we counted the number of organoids per well that were located between the three dots. We found that this normalization worked well for conditions with similar organoid size and viability. For substrate oxidation assays in which organoid samples from the same lineage were treated with different inhibitors, samples were instead normalized by percentage of OCR to correct for differences in organoid number per well, a normalization directly provided by Agilent software (https://seahorseanalytics.agilent.com).

### Preparation of single-cell suspensions from intestinal and colonic mucosa for cell sorting and FACS-based immunophenotyping

#### Intestinal preparation

Crypts were isolated as described above. After spinning, crypts were incubated in DMEM/F-12 medium (D8437, Sigma-Aldrich) supplemented with 0.8 U ml^−1^ dispase (17105041, Life Technologies, Thermo Fisher Scientific) and 1 mg ml^−1^ DNase (04716728001, Roche). Cells were incubated in 2 ml of ‘digestion solution’ for 10–15 min at 37 °C, vortexing the samples every 2 min for 30 s. After 10 min, a 20-µl sample was taken and observed under the microscope to check single-cell dissociation. Digestion was stopped by adding 10 ml of fetal bovine serum. Cells were then filtered through a 70-μm mesh, spun down at 290*g* for 5 min and resuspended in MACS buffer (0.5% BSA and 2 mM EDTA in Ca^2+^/Mg^2+^-free PBS). GFP^high^ (ISCs), GFP^low^ (TA cells), GFP^−^ high side scattering and forward scattering (Paneth cells) and the villus fraction (first fraction during mechanical cell extraction) were isolated from *Lgr5-EGFP-IRES-creERT2* mice and sorted using a Sony MA900 cell sorter.

#### Colon preparation

For immunophenotyping of the lamina propria and the muscle layer in the colon of control and DSS-treated mice, samples were prepared as previously reported^67^. In brief, the entire colon was dissected, opened longitudinally and washed in 1× HBSS (Gibco, 14025-092). The colon was chopped into pieces of 0.5–1 cm and crypts were mechanically dissociated using 8 mM EDTA. The remaining pieces of colon were transferred to digestion mix with dispase and DNAse. Immune cells were further sedimented by centrifugation and immunophenotyping analysis was performed as described below.

### FACS-based immunophenotyping

For multiparametric flow-cytometry analysis, cell suspensions were stained with LIVE/DEAD fixable viability dye (1: 500, Invitrogen, R37601) for 30 min in PBS at 4 °C. After this, cells were washed, incubated with Fc block (1: 200, BD Biosciences, 564219) in FACS buffer for 15 min at 4 °C and then stained with a cocktail of conjugated antibodies (see below) for 30 min on ice. After staining, cells were washed three times with FACS buffer and fixed using BD Cytofix/Cytoperm (Thermo Fisher Scientific, 544772) for 20 min at 4 °C, washed again and stored for analysis. Samples were analysed in a BD LSR Fortessa with five lasers, and gates were set by fluorescence-minus-one controls.

The following antibodies were used for flow-cytometry analysis: AF700 CD45 (BioLegend, 103128, clone 30-F11, 1:200), BUV395 CD11b (BD Biosciences, 563553, clone M1/70, 1:200), PE F4/80 (BioLegend, 123110, clone BM8, 1:200), BV605 Ly6G (BD Bioscience, 563005, clone 1A8, 1:200), APC Cy7 Ly6c (BioLegend, 128026, clone HK1.4, 1;200), APC MHCII (BioLegend, 107614, clone M5/114.15.2, 1:200), BV710 CD206 (BioLegend, 141727, clone C068C2, 1:200) and BV650 CD86 (BioLegend, 105035, clone GL-1).

### Faecal LCN2 content

Faecal samples were collected longitudinally and analysed for LCN2 levels using an ELISA according to the manufacturer’s instructions (Abcam, ab199083). Frozen faecal samples were reconstituted in PBS containing 0.1% Tween 20 at a concentration of 100 mg ml^−1^. The samples were vortexed for 20 min to achieve a homogeneous suspension and then centrifuged at 12,000 rpm for 10 min at 4 °C. The clear supernatants were collected and stored at −20 °C until analysis. Measurements were normalized to the weight of the faecal samples.

### Immunofluorescence

#### Mouse tissues

Mouse tissues were fixed overnight at 4 °C in 10% formalin before paraffin embedding. Five-micrometre sections were deparaffinized and rehydrated with Histo-Clear (Thermo Fisher Scientific, National Diagnostics) and an alcohol series and subjected to antigen retrieval by boiling in citrate antigen retrieval buffer (Vector). Slides were blocked in PBS with 5% BSA, and primary antibody staining was performed in blocking buffer + 0.02% Triton X-100 overnight at 4 °C. The following primary antibodies were used: chicken anti-GFP (1:500, Abcam 13970), mouse anti-Ki67 (1:500, BD, 550609), rabbit anti-p53 (1:500, NCL-L-p53-CM5p, Leica Biosystems), rabbit anti-5hmC (1:500, Active Motif, 39769), mouse anti-β-catenin (1:200, BD, 610153), rabbit anti-OGDH (1:100, Proteintech, 15212-1-AP), rabbit anti-VDAC (1:100, Abcam, ab15895), goat anti-ACE2 (1:100, Thermo Fisher Scientific, PA5-47488), rabbit anti-lysozyme (1:500, Thermo Fisher Scientific, MA5-32154), rabbit anti-BrdU (1:100, Abcam, ab6326), rabbit anti-cleaved caspase 3 (1:200, Cell Signaling, 9664S), mouse anti-HNF4α (1:100, Thermo Fisher Scientific, MA1-199), mouse anti-TET1 (1:100, Thermo Fisher Scientific, MA5-16312), rabbit anti-TET2 (1:100, Thermo Fisher Scientific, PA5-85488), rabbit anti-TET3, (1:100, Thermo Fisher Scientific, PA5-31860), rat anti-CD8 (1:200, 14-0808-82, Thermo Fisher Scientific) and rat anti-CD4 (1:100, Thermo Fisher Scientific, 14-9766-82). Primary antibodies were detected with the following fluorescently conjugated secondary antibodies: goat anti-chicken AF488 (Life Technologies A-11039, 1:1,000), goat anti-rabbit AF488 (Life Technologies A-32723, 1:1,000), goat anti-rabbit AF594 (Life Technologies A-11037, 1:1,000), goat anti-mouse AF488 (Life Technologies, A-32723, 1:1,000), goat anti-mouse AF594 (Life Technologies, A-11032, 1:1,000), goat anti-rat AF488 (Life Technologies, A-11006, 1:1,000) and goat anti-rat 594 (Life Technologies, A-11007, 1:1,000). All secondary antibodies were diluted in blocking buffer + 0.02% Triton X-100 and incubated for one hour at room temperature. Slides were then washed with PBS and nuclei were counterstained with PBS containing DAPI and mounted under coverslips with ProLong Gold (Life Technologies).

#### Human samples

Samples used in human studies were obtained from TissueArray (https://www.tissuearray.com/). Specifically, tissue microarrays (TMAs) CO809b, CO246, CO245a were used. Immunofluorescence staining was performed as described above, using the same primary and secondary antibodies.

#### Organoid immunofluorescence

Organoids were recovered using Cell Recovery Solution (354253, Corning BD) and fixed with 4% paraformaldehyde (PFA) for 20 min at room temperature (18–21 °C). Next, the samples were passed through an ethanol series (70%, 96% and 100%) and embedded in paraffin. Immunohistochemistry and immunofluorescence were performed using standard techniques as described above, using the same primary and secondary antibodies.

#### Image acquisition and analysis

Images were acquired with a Zeiss AxioImager microscope using Axiovision software. Five to ten images per slide were obtained. Quantification was performed either by counting the number of positive cells per crypt or villus (at least 50 crypts and 100 villi were quantified) or by calculating the percentage of the positive area using the Color Deconvolution plug-in in ImageJ v.1.7 software. Additional macros were developed by the authors to quantify immunofluorescence images.

### Multiplex immunofluorescence in human and mouse FFPE tissues

Multiplex immunofluorescence imaging was performed using the Comet Lunaphore platform^68^ for mouse tissues and the CellDive platform^69^ for human TMAs of formalin-fixed paraffin-embedded (FFPE) tissues. Tissue processing was done as described above (‘Immunofluorescence’ section), with the key difference that antigen retrieval included the use of two buffers. Specifically, after deparaffinization, slides were boiled in citrate antigen retrieval buffer (pH 6.0, Vector Labs H-3300-250) and then directly transferred to Tris-EDTA unmasking solution (pH 9.0, Vector Labs H-3301-250) and boiled again. This dual antigen retrieval method improved antigen unmasking in our samples, enabling the combination of antibodies that work with either citrate or EDTA buffers. After antigen retrieval, the slides were loaded onto the respective platforms. For details on the antibody panels and staining conditions, see Supplementary Tables 4 (COMET Lunaphore mouse panel) and 5 (CellDive human panel). For CellDive multiplex immunofluorescence, the first round of staining was performed using primary and secondary antibodies. In most cases, subsequent rounds used primary conjugated antibodies. In addition, a stripping step was performed as previously described^70^, allowing for a second round of primary and secondary antibody staining on the same TMAs. The HNF4α antibody was self-conjugated according to the manufacturer’s instructions (https://www.thermofisher.com/order/catalog/product/A20186).

### smFISH

smFISH analysis was performed as previously described, with slight modifications in the protocol^71^.

#### Probe design for multiplex smFISH

We built on published software^72,73^ to design custom panels for smFISH. This design strategy relies on the precomputation of all possible 30-mer sequences found in mouse cDNAs (Ensembl GRCm38.p6), augmented with coding sequences of fluorescent proteins engineered into our mouse model. We excluded pseudogenes from the potential pool of mRNAs to design probes for. We computed multiple scores for each 30-mer, including *T*_m_, GC content and potential for hybridization with ribosomal RNAs (rRNAs) and transfer RNAs (tRNAs). We used the following criteria for including a 30-mer in our candidate probe set: GC content 43–63% and *T*_m_ (66–76 °C), excluding 30-mers that contain at least a 15-mer present in an rRNA or a tRNA. In addition, we computed expression-informed penalties to estimate the specificity of each candidate probe. We adapted published software^72,73^ to include single-cell information into the estimation of specificity scores. We reasoned that incorporating single-cell information would decrease the chances of selecting probes with off-target binding to highly expressed genes in rare cell populations. To do so, we used the published single-cell data used in figure 1 in ref. ^26^. Following suggested parameters from the original MERFISH publications^72,73^, we considered 30-mers with a specificity score greater than 0.75 as candidates for our panels. We aimed to select 92 non-overlapping probes per gene. Whenever this was not possible owing to transcript length, homology to other genes or other sequence properties, we allowed a maximum overlap of 20 bp between probes. Probe sequences can be found in Supplementary Table 3.

#### Sample preparation

‘Swiss-rolled’ intestines were positioned in a cassette and fixed with 4% PFA 1× PBS solution for four hours at 4 °C. Cassettes were then transferred to 4% PFA and 30% sucrose in 1× PBS and incubated overnight at 4 °C. To preserve villus structures, cassettes were transferred for four hours to 30% sucrose and 50% OCT for three hours at room temperature and finally embedded in Tissue Plus OCT Compound (Fisher Healthcare, 4585) in a cryomold (Tissue-Tek, 4557). Moulds were placed on dry ice until all OCT was frozen, and intestinal samples were stored at −80 °C.

#### Coverslip preparation

Coverslips for smFISH staining were prepared as previously described^71^. In brief, 40-mm-diameter coverslips (Bioptechs, 0420-0323-2) were cleaned by immersing them in a 1:1 mix of 37% HCl and methanol at room temperature for 30 min. Coverslips were then washed with Milli-Q water, washed once with 70% ethanol and then gently dried with nitrogen gas. Cleaned coverslips were submerged in 0.1% (v/v) triethylamine (Millipore, TX1200) and 0.2% (v/v) allyltrichlorosilane (Sigma, 107778) in chloroform for 30 min at room temperature. They were washed once with chloroform and once with 100% ethanol, then dried using nitrogen gas. Coverslips were stored long term in a desiccated chamber at room temperature.

#### Poly-lysine coating of coverslips

To prepare coverslips for staining individual samples, pre-treated coverslips were coated with 0.1 mg ml^−1^ poly-d-lysine (Thermo Fisher Scientific, A3890401) for one hour at room temperature. Next, they were washed once with 1× PBS, and three times with nuclease-free water. After that, they were left to dry for at least two hours before sectioning the tissue.

#### Tissue sectioning, fixation and permeabilization for smFISH staining

Tissue sections of 10-μm thickness were mounted into poly-d-lysine-coated coverslips. Coverslips were dried for 5–10 min at 50 °C and placed on dry ice until completion of sectioning of all samples. Next, plates with coverslips were transferred to ice, and treated with 3 ml 1× PBS, followed by fixation at room temperature with 4% PFA 1× PBS for 10 min. Coverslips were then washed three times with 1× PBS and maintained at 4 °C overnight in ice-cold 70% ethanol for permeabilization.

#### Pre-staining treatment of permeabilized tissues

After overnight incubation, coverslips were rehydrated with 1× PBS on ice for 10 min. To bleach endogenous fluorescence of lineage reporters and reduced autofluorescence from lysozyme granules, tissues were incubated with 3% hydrogen peroxide (Thermo Fisher Scientific, H325-500), 1:600 37% HCl (v/v) 1× PBS and placed under a heat lamp for one hour at room temperature. They were then washed twice with 1× PBS and once with 2× SSC. Next, they were treated with digestion solution (pre-warmed at 37 °C) containing a final concentration of 20 μg ml^−1^ proteinase K (Sigma, 3115836001) in 2× SSC solution, and incubated at 37 °C for 10 min. Next, coverslips were washed three times with 2× SSC and treated with pre-hybridization solution (30% formamide (Thermo Fisher Scientific, AM9344) and 2× SSC) and incubated for at least three hours at 37 °C.

#### Staining with primary probes

Primary probes were diluted to 100 nM per probe in 3H staining buffer (30% formamide, 10% dextran sulfate (Sigma-Aldrich, D8906-50G), 1 mg ml^−1^ yeast tRNA (Thermo Fisher Scientific, 15401029) and 2× SSC). In addition, 2 μM anchor probe was added to the staining solution containing specific probes. A 100-µl droplet of this solution was then added to coverslips after their pre-hybridization incubation, then the coverslips were placed on a 15-cm dish with a wet Kimwipe used as a humidity buffer and incubated at 37 °C for 36–48 h. Next, post-hybridization wash buffer (30% formamide and 2× SSC) was pre-heated to 37 °C. Coverslips were washed twice with post-hybridization wash buffer at 47 °C for 30 min. Finally, coverslips were transferred to 2× SSC solution and maintained at 4 °C until the next step.

#### Gel embedding and digestion

Samples were embedded on a thin layer of polyacrylamide gel, to allow subsequent tissue clearing through digestion of protein and lipids. The gel solution was composed of 4% (v/v) 19:1 acrylamide/bis-acrylamide (Bio-Rad, 1610144), 60 mM Tris⋅HCl pH 8 (Invitrogen, 15568-025) and 0.3 M NaCl (Boston BioProducts, R-244), supplemented with the polymerizing agents ammonium persulfate (Sigma, 09913) and TEMED (Sigma, T7024) at final concentrations of 0.03% (w/v) and 0.15% (v/v), respectively, and polymerized as previously described^71^. Polymerization was complete after two hours at room temperature. Next, gel-embedded coverslips were transferred to a 6-cm tissue culture dish with 2× SSC. Gel-embedded samples were treated overnight at 37 °C with digestion solution: 2% SDS (Invitrogen, AM9822), 0.25% Triton X-100 (Acros Organics, 327371000) and a 1:100 dilution of proteinase K (NEB, P8107S) in 2× SSC. After overnight digestion, samples were washed for 30 min with 2× SSC and gentle agitation.

#### Staining with secondary probes

We used readout probes consisting of a 20-bp oligonucleotide conjugated to a fluorophore (Alexa Fluor 488, Cy3B, Cy5 or Alexa Fluor 750) through a disulfide bond. Fluorescent conjugated probes were purchased from Bio-Synthesis. The secondary staining solution was composed of 5% ethylene carbonate (Sigma-Aldrich, E26258-100G) in 2× SSC. The secondary staining solution was supplemented by a secondary readout probe for each fluorescent colour at a final concentration of 3 nM, and with DAPI at a final concentration of 1 μM. Secondary staining was performed following the same procedure as the primary staining step, with the exception that it was done for 20 min at room temperature, covering samples with aluminium foil. After hybridization, samples were washed once with 10% ethylene carbonate in 2× SSC for 20 min with gentle agitation, and three times with 2× SSC for 5 min per wash.

#### Iterative smFISH imaging

Iterative smFISH imaging was performed as previously described^71^. Combinations of readout sequences and target mRNA species are provided in Supplementary Table 3.

#### smFISH image processing and analysis

To collapse *z*-stacks into a single two-dimensional image, maximum projection images were generated using the Nikon Elements software’s maximum projection function. After each round of FISH imaging, we took an additional image with cleaved fluorophores to capture the background signal for each channel. Because the microscope has unequal sensitivity to the five fluorophores, we also imaged each fluorophore’s flat field to capture its bias. We corrected raw FISH images by subtracting the background signal of each gene and then dividing by the flat-field bias of the conjugated fluorophore, then thresholding to 0 to correct any negative-valued pixels. Additional alignment was performed using DAPI.

### Transmission electron microscopy

Duodenal samples from C57Bl/6 mice were collected and prepared as described above. One- to two-millimetre intestinal samples were fixed in 2% glutaraldehyde, 4% PFA and 2 mM CaCl_2_ in 0.1 M sodium cacodylate buffer, pH 7.2, at room temperature for more than one hour, dehydrated in an acetone series, post-fixed in 1% osmium tetroxide and processed for Eponate 12 (Ted Pella) embedding. Ultrathin sections (65 nm) were cut, post-stained with uranyl acetate and lead citrate and imaged in a Tecnai 12 electron microscope (FEI), operating at 120 kV and equipped with an AMT BioSprint29 digital camera (AMT Imaging). Mitochondrial quantification was performed as previously described^74^.

### Immunoblotting

Immunoblotting was performed in mouse ES cells containing a doxycycline-inducible GFP-coupled shRNA^30,33^. The genotypes used were sh*Ogdh*_2081, sh*Ogdh*_346 (ref. ^1^) and sh*Ren*_731. Mouse ES cells were treated with doxycycline for 72 h. In brief, the supernatant was removed, and the cells were washed three times with PBS. Mouse ES cells were then lysed using RIPA lysis buffer (Sigma-Aldrich, R0278) supplemented with NaF (1 mM), Na_4_P_2_O_7_ (20 mM) and Na_3_VO_4_ (2 mM). The lysates were incubated for 15 min on ice and subsequently clarified by centrifugation at 4 °C and 10,000*g*. The protein concentration was determined using the Pierce BCA protein assay kit (23225, Thermo Fisher Scientific) following the manufacturer’s instructions. Lysates were concentrated to a final concentration of 1 mg ml^−1^ by boiling the appropriate amount of protein lysate with 2× Laemmli buffer (4% SDS, 20% glycerol, 10% 2-mercaptoethanol and 0.004% bromophenol blue in 0.2 M Tris-HCl, pH 7) at 90 °C for 10 min. Twenty micrograms of protein lysates were loaded onto SDS–PAGE gels and transferred to 0.2-μm nitrocellulose membranes (LI-COR Biosciences, 926-31090). The membranes were blocked with 5% blotting-grade blocker (non-fat dry milk, 170-6404, Bio-Rad) in Tris-buffered saline containing 1% Tween 20 (TBS-T) for one hour at room temperature. Then the membranes were incubated overnight at 4 °C in TBS-T with 3% sodium azide with rabbit anti-OGDH antibody (1:500, Proteintech, 15212-1-AP). After washing the membranes three times with TBS-T for 10 min each at room temperature, they were incubated with secondary anti-rabbit antibody (1:5,000, Cell Signaling, 7074S) in 1% blotting-grade blocker. The protein bands were visualized using enhanced chemiluminescence (ECL) detection reagent (Cell Signaling, 6883P3) according to the manufacturer’s instructions.

### ChIP in intestinal crypts

Intestinal crypts were isolated and cross-linked for 10 min at room temperature in 1% formaldehyde. Cross-linking reactions were stopped by adding 1.25 M glycine to a final concentration of 125 mM. The crypts were then centrifuged for 10 min at 4 °C and washed in cold PBS. The cells were lysed with 1 ml lysis buffer 1 (50 mM HEPES-KOH, pH 7.5, 140 mM NaCl, 1 mM EDTA, 10% glycerol, 0.5% NP-40, 0.25% Triton X-100 and 1× protease inhibitor) followed by centrifugation at 4 °C. The pellet was resuspended in lysis buffer 2 (10 mM Tris-HCl, pH 8.0, 200 mM NaCl, 1 mM EDTA, 0.5 mM EGTA and 1× protease inhibitors). Finally, the pellets were resuspended in 1 ml lysis buffer 3 (10 mM Tris-HCl, pH 8.0, 100 mM NaCl, 1 mM EDTA, 0.5 mM *N*-lauroylsarcosine and 1× protease inhibitors) and 100 μl 10% Triton X-100 was added. The samples were sonicated for 10 min using an E220 Focused Ultrasonicator (Covaris, PN 500239). Samples were sonicated using milliTube 1 ml with AFA fibre (Covaris, PN 520130) under flowing conditions (140 pip, 5% duty cycle, 200 CBP). The soluble fraction was quantified using the Bradford assay, and 400 μg of the soluble fraction was used for immunoprecipitation of the transcription factors HNF4α (1 μg, Thermo Fisher Scientific, MA1-199), and SMAD4 (1 μg, Cell Signaling, 46535) with rabbit IgG (1 μg, Cell Signaling, 2729S) and mouse IgG (1 μg, Santa Cruz Biotechnology, sc-2025) used as a control. The chromatin and antibody mixtures were incubated overnight at 4 °C in a total volume of 500 μl. The immunoprecipitated mixture was then washed sequentially with a Triton dilution buffer (1% Triton X-100, 2 mM EDTA pH 8, 150 mM NaCl and 20 mM Tris-HCl pH 8.1), mixed micelle wash buffer (1% Triton X-100, 5 mM EDTA pH 8, 150 mM NaCl, 20 mM Tris-HCl pH 8.1, 5% sucrose, 0.2% NaN_3_ and 0.2% SDS), buffer 500 (0.1% w/v deoxycholic acid, 1 mM EDTA pH 8, 50 mM HEPES pH 7.5, 500 mM NaCl, 1% Triton X-100, 0.2% NaN_3_) and a LiCl wash buffer (0.5% w/v deoxycholic acid, 1 mM EDTA pH 8, 250 mM LiCl, 0.5% v/v NP-40, 10 mM Tris-HCl and 0.2% NaN_3_). The samples were de-cross-linked, and the DNA was extracted using phenol, chloroform and isoamyl alcohol mixtures, washed with 80% ethanol and resuspended in 200 μl TE buffer. qRT–PCR was performed using specific primers (Supplementary Table 2).

### Statistical analysis and data representation

Before performing any statistical test, we tested for normal distribution using the D’Agostino–Pearson test. For continuous variables, we used the *t*-test, Mann–Whitney *U* test, one‐way ANOVA or Friedman’s test. For categorical variables, we used the chi‐squared test or Fisher’s exact test. The Mantel–Cox test was used to analyse the Kaplan–Meier survival of mice. If significant differences by one-way ANOVA were found, group-wise comparisons were done using Tukey’s multiple comparisons test. If significant differences by Friedman’s test were found, Dunn’s multiple comparisons test was used. The predictive value of *Ogdh* expression in enterocytes, ISCs and Paneth cells was evaluated by examining the area under the receiver operating characteristic (ROC) curve with a confidence interval of 95%. All statistical tests were considered statistically significant when *P* was less than 0.05. Statistical significance in figures is summarized as follows: *P ≤ 0.05, **P ≤ 0.01, ***P ≤ 0.001 and ****P ≤ 0.0001 between the means of a minimum of three samples. Results are expressed as mean ± s.e.m.

The immunofluorescence, H&E, other stainings and IHC data shown are representative of at least three independent mice. Quantification of immunofluorescence, H&E and other stainings was performed for more than 50 crypts or 100 villi per mouse in at least 3 independent mice. For in vitro experiments, at least three independent experiments were performed. For the in vivo experiments, at least five mice per group were used.

### Reporting summary

Further information on research design is available in the Nature Portfolio Reporting Summary linked to this article.

## Online content

Any methods, additional references, Nature Portfolio reporting summaries, source data, extended data, supplementary information, acknowledgements, peer review information; details of author contributions and competing interests; and statements of data and code availability are available at 10.1038/s41586-025-09097-6.

## Supplementary information

Supplementary Information Supplementary Figs. 1–3, showing the uncropped blots and gating strategies.

Reporting Summary

Supplementary Table 1 Statistical analysis scRNA-seq data - Statistical summary and significance values for scRNA-seq analysis.

Supplementary Table 2 qPCR primers - List of primers used for qPCR experiments.

Supplementary Table 3 smFISH-probes - Sequences and details of probes used for smFISH.

Supplementary Table 4 Panel for Comet/ Lunaphore mouse FFPE tissue - List of markers and antibodies used for multiplexed imaging on formalin-fixed paraffin-embedded (FFPE) mouse tissues.

Supplementary Table 5 Panel for CellDive human FFPE tissue microarray - List of markers and antibodies used for multiplexed imaging on human FFPE tissue microarrays using CellDive technology.

Supplementary Table 6 Plasmids maps and shRNA/ cDNA sequences - Detailed maps of plasmids and sequences for shRNA and cDNA constructs used in the study.

Supplementary Table 7 Loci-targeted 5mC/ 5hmC primers - List of primers designed for targeted analysis of 5mC and 5hmC modifications at specific genomic loci.

Supplementary Table 8 Gene signatures - Curated gene signatures used in transcriptomic analyses, including relevant pathway annotations.

## Source data

Source Data Fig. 1

Source Data Fig. 3

Source Data Fig. 4

Source Data Fig. 5

Source Data Extended Data Fig. 1

Source Data Extended Data Fig. 2

Source Data Extended Data Fig. 3

Source Data Extended Data Fig. 4

Source Data Extended Data Fig. 5

Source Data Extended Data Fig. 6

Source Data Extended Data Fig. 7

Source Data Extended Data Fig. 9

Source Data Extended Data Fig. 10

## Data Availability

The data supporting the findings of this study have been deposited in the Gene Expression Omnibus (GEO) under the accession number GSE293287.  Source data are provided with this paper.

## Code availability

No custom code or mathematical algorithm was used in this study that is central to the conclusions.
