# Publisher Correction: Metabolic adaptations direct cell fate during tissue regeneration

**DOI:** 10.1038/s41586-025-09294-3

**Published:** 2025-07-01

**Authors:** Almudena Chaves-Perez, Scott E. Millman, Sudha Janaki-Raman, Yu-Jui Ho, Clemens Hinterleitner, Valentin J. A. Barthet, John P. Morris, Francisco M. Barriga, Jose Reyes, Aye Kyaw, H. Amalia Pasolli, Dana Pe’er, Craig B. Thompson, Lydia W. S. Finley, Justin R. Cross, Scott W. Lowe

**Affiliations:** 1 Cancer Biology and Genetics Program, Memorial Sloan Kettering Cancer Center, New York, NY USA; 2 Donald B. and Catherine C. Marron Cancer Metabolism Center, Memorial Sloan Kettering Cancer Center, New York, NY USA; 3 Computational and Systems Biology Program, Memorial Sloan Kettering Cancer Center, New York, NY USA; 4 Electron Microscopy Resource Center, The Rockefeller University, New York, NY USA; 5 Cell Biology Program, Memorial Sloan Kettering Cancer Center, New York, NY USA

**Keywords:** Stem-cell differentiation, Intestinal stem cells, Ulcerative colitis, Energy metabolism

Correction to: *Nature* 10.1038/s41586-025-09097-6 Published online 11 June 2025

In the version of the article initially published, in the top panel of Fig. 1b, the labels “ISCs” and “Abs” were switched and have now been amended so that the labels read (from top to bottom) “ISCs, Abs, Sec” in the HTML and PDF versions of the article.

