# Supplementary material for: Metabolic adaptations direct cell fate during tissue regeneration
Source: Nature. 2025 Jun 11;643(8071):468–77. doi: 10.1038/s41586-025-09097-6 (PMC12240837; doi:10.1038/s41586-025-09097-6)
Supplement: Supplementary file 1 — Supplementary Figs. 1–3, showing the uncropped blots and gating strategies. [file 41586_2025_9097_MOESM1_ESM.pdf]

nature portfolio

https://doi.org/10.1038/s41586-025-09097-6

Supplementary information

Metabolic adaptations direct cell fate during tissue regeneration

In the format provided by the authors and unedited

Supplementary information

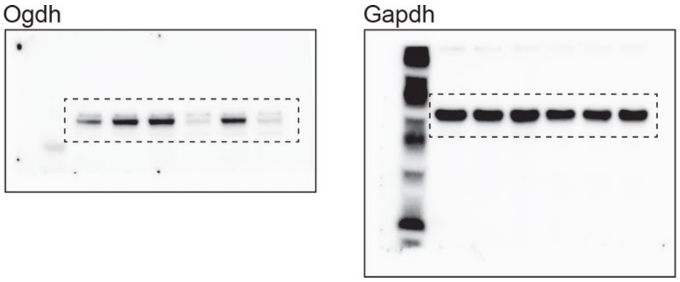

Supplementary Figure 1| Western blot showing Ogdh downregulation in mESCs. Raw files for the western blot shown in **Extended Data Figure 4d**. GAPDH was run on the same gel and used as loading control.

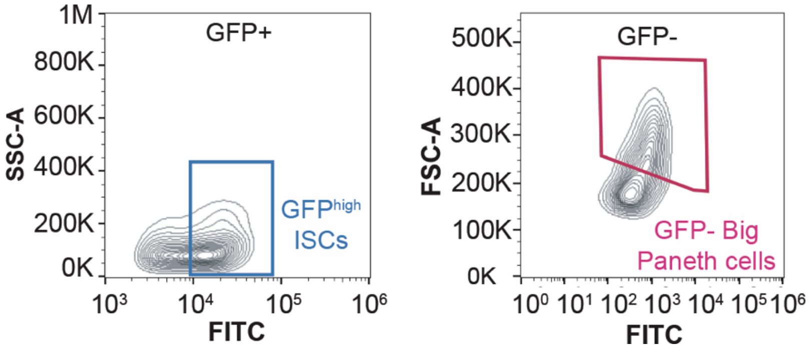

Supplementary Figure 2| Gating strategy to isolate ISCs from Lgr5-EGFP mice. Gating strategy for **Figure 1b**, showing the sorting of ISCs (FITC^{high}) and Paneth cells (FITC−/FSC-A^{high}) from isolated, single-cell–dissociated crypts of Lgr5-EGFP reporter mice.

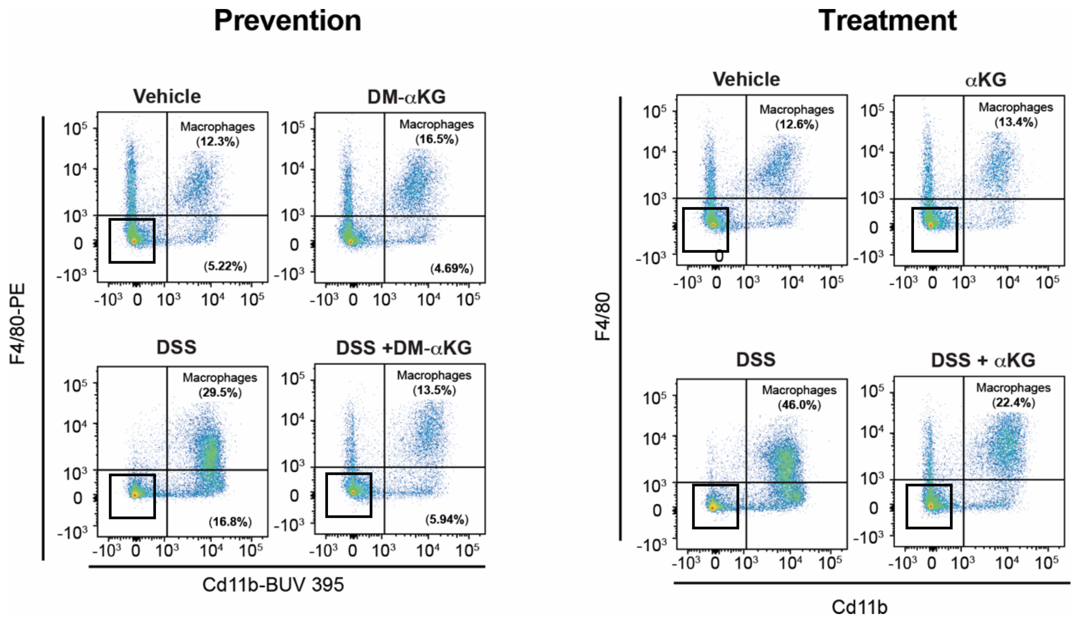

Supplementary Figure 3| Gating strategy to analyze immune infiltration in colitis mice. Gating strategy for **Extended data Figure 10h, I, p and q**, showing macrophages ($CD11b^{high}/F4\_80^{high}$) and the quadrant used to identify other myeloid populations ($CD11b^{high}/F4\_80^{low}$). $CD11b^{high}/F4\_80^{low}$ cells were further subdivided as granulocytes (Ly6G+/Ly6C+) and monocytes (Ly6G-/Ly6C+) in both prevention and treatment settings with αKG in DSS-treated mice.
