# Supplementary material for: Metabolic adaptations direct cell fate during tissue regeneration
Source: Nature. 2025 Jun 11;643(8071):468–77. doi: 10.1038/s41586-025-09097-6 (PMC12240837; doi:10.1038/s41586-025-09097-6)
Supplement: Supplementary file 2 — Reporting Summary [file 41586_2025_9097_MOESM2_ESM.pdf]

nature portfolio

Corresponding author(s): Dr. Scott Lowe

Last updated by author(s): Mar 7, 2025

Reporting Summary

Nature Portfolio wishes to improve the reproducibility of the work that we publish. This form provides structure for consistency and transparency in reporting. For further information on Nature Portfolio policies, see our Editorial Policies and the Editorial Policy Checklist.

Statistics

For all statistical analyses, confirm that the following items are present in the figure legend, table legend, main text, or Methods section.

n/a	Confirmed	
☐	☒	The exact sample size (*n*) for each experimental group/condition, given as a discrete number and unit of measurement
☐	☒	A statement on whether measurements were taken from distinct samples or whether the same sample was measured repeatedly
☐	☒	The statistical test(s) used AND whether they are one- or two-sided<br>*Only common tests should be described solely by name; describe more complex techniques in the Methods section.*
☐	☒	A description of all covariates tested
☐	☒	A description of any assumptions or corrections, such as tests of normality and adjustment for multiple comparisons
☐	☒	A full description of the statistical parameters including central tendency (e.g. means) or other basic estimates (e.g. regression coefficient) AND variation (e.g. standard deviation) or associated estimates of uncertainty (e.g. confidence intervals)
☐	☒	For null hypothesis testing, the test statistic (e.g. *F*, *t*, *r*) with confidence intervals, effect sizes, degrees of freedom and *P* value noted<br>*Give P values as exact values whenever suitable.*
☒	☐	For Bayesian analysis, information on the choice of priors and Markov chain Monte Carlo settings
☐	☒	For hierarchical and complex designs, identification of the appropriate level for tests and full reporting of outcomes
☐	☒	Estimates of effect sizes (e.g. Cohen's *d*, Pearson's *r*), indicating how they were calculated

Our web collection on statistics for biologists contains articles on many of the points above.

Software and code

Policy information about availability of computer code

Data collection	Multiplexed immunofluorescence data were acquired using the Cell DIVE™ platform (Leica Microsystems), with image acquisition, processing, and quantification performed using Cell DIVE™ software (version 4.0). COMET™ (Lunaphore) experiments were conducted following the manufacturer's protocols, with staining, imaging, and analysis performed using COMET™ software (version 1.7).
Data analysis	No software was usedFurther image processing and quantification were carried out using QuPath (version 0.4.3) (qupath.github.io), ImageJ (version 1.53t) (imagej.net, download here), and Fiji (version 2.9.0) (imagej.net). Additionally, Axiovision (version 4.9.1) was used for the analysis of conventional immunofluorescence images.<br><br>Flow cytometry data were analyzed using FlowJo (version 10.9.0) (flowjo.com).<br><br>All experimental conditions, including antibody panels, dilutions, incubation times, and imaging parameters, are detailed in the Methods section and corresponding tables.

For manuscripts utilizing custom algorithms or software that are central to the research but not yet described in published literature, software must be made available to editors and reviewers. We strongly encourage code deposition in a community repository (e.g. GitHub). See the Nature Portfolio guidelines for submitting code & software for further information.

Data

Policy information about availability of data

All manuscripts must include a data availability statement. This statement should provide the following information, where applicable:

- Accession codes, unique identifiers, or web links for publicly available datasets
- A description of any restrictions on data availability
- For clinical datasets or third party data, please ensure that the statement adheres to our policy

The data supporting the findings of this study have been deposited in the Gene Expression Omnibus (GEO) under the accession number GSE293287. The dataset is publicly available at https://www.ncbi.nlm.nih.gov/geo/query/acc.cgi?acc=GSE293287. Additional data are available from the corresponding author upon reasonable request.

Research involving human participants, their data, or biological material

Policy information about studies with human participants or human data. See also policy information about sex, gender (identity/presentation), and sexual orientation and race, ethnicity and racism.

Reporting on sex and gender	N/A
Reporting on race, ethnicity, or other socially relevant groupings	N/A
Population characteristics	N/A
Recruitment	N/A
Ethics oversight	N/A

Note that full information on the approval of the study protocol must also be provided in the manuscript.

Field-specific reporting

Please select the one below that is the best fit for your research. If you are not sure, read the appropriate sections before making your selection.

☒ Life sciences ☐ Behavioural & social sciences ☐ Ecological, evolutionary & environmental sciences

For a reference copy of the document with all sections, see nature.com/documents/nr-reporting-summary-flat.pdf

Life sciences study design

All studies must disclose on these points even when the disclosure is negative.

Sample size	To determine the sample size for mouse experiments, we conducted a statistical power analysis. This analysis took into account the expected effect size, an alpha level of 0.05, a desired power level of 0.80, and population variability estimated from previous studies. Using these parameters, we calculated the required sample size to ensure the study is adequately powered to detect the anticipated effects, utilizing power analysis software and relevant statistical formulas. Based on this criteria, sample size varies from 5 to 8 mice per conditions. Several experiments included additional mice, even after achieving the necessary statistical power (e.g., DSS treatment), to ensure the reproducibility of the data.
Data exclusions	No data points were excluded from the analysis
Replication	In the case of mouse experiments, results were confirmed in at least three independent experiments.<br>For metabolomic data, due to the difficulty of plating the same number of organoids per mouse, crypts from five mice were pooled and plated in triplicate. Each triplicate was run and analyzed independently.<br><br>For Seahorse assays, organoids from independent mice were plated, with more than three mice used per condition. All data were reproducible across all attempts. All the attempts were succesfull.
Randomization	For DSS treatment, the groups were randomized based on body weight. At the initial time points of each experiment, all groups had mice with the same average body weight.<br><br>For all experiments, except those involving DSS treatment, mice were allocated into experimental groups based on their specific genotype. Within each genotype, mice were randomly assigned to the corresponding treatment groups. This random allocation ensured unbiased treatment assignment and minimized potential confounding variables. Since the experiments were genotype-specific, no additional covariates were controlled for in this context.

Blinding

Investigators were blinded to group allocation and data analysis.

Reporting for specific materials, systems and methods

We require information from authors about some types of materials, experimental systems and methods used in many studies. Here, indicate whether each material, system or method listed is relevant to your study. If you are not sure if a list item applies to your research, read the appropriate section before selecting a response.

Materials & experimental systems

n/a	Involved in the study
☐	☒ Antibodies
☒	☐ Eukaryotic cell lines
☒	☐ Palaeontology and archaeology
☐	☒ Animals and other organisms
☒	☐ Clinical data
☒	☐ Dual use research of concern
☒	☐ Plants

Methods

n/a	Involved in the study
☒	☐ ChIP-seq
☐	☒ Flow cytometry
☒	☐ MRI-based neuroimaging

Antibodies

Antibodies used

The following primary antibodies were used: chicken anti-GFP (1:500, Abcam 13970, https://www.abcam.com/en-us/products/primary-antibodies/gfp-antibody-ab13970), mouse anti-Ki67 (1:500, BD, 550609, Clone 56 (RUO) https://www.bdbiosciences.com/en-es/products/reagents/flow-cytometry-reagents/research-reagents/single-color-antibodies-ruo/purified-mouse-anti-ki-67.550609?tab=product_details), rabbit anti-p53 (1:500, NCL-L-p53-CM5p, Leica Biosystems, https://shop.leicabiosystems.com/es-es/ihc-ish/ihc-primary-antibodies/pid-p53-protein-cm5), rabbit anti-5hmC (1:500, Active Motif, 39769, https://www.activemotif.com/catalog/details/39769), mouse anti-β-catenin (1:200, BD, 610153, clone 14/ catenin (RUO), https://www.bdbiosciences.com/en-es/products/reagents/microscopy-imaging-reagents/immunofluorescence-reagents/purified-mouse-anti-catenin.610153?tab=product_details), rabbit anti-Ogdh (1:100, Proteintech, 15212-1-AP, https://www.ptglab.com/products/OGDH-Antibody-15212-1-AP.htm?srsltid=AfmBOoohrwP797YeCUvojyQLmEek1jzAUChFjXOtfu6_Gs32qL0SiaKC), rabbit anti-VDAC (1:100, Abcam, ab15895, https://www.abcam.com/en-us/products/primary-antibodies/vdac1-porin-vdac2-vdac3-antibody-mitochondrial-loading-control-ab15895), goat anti-Ace2 (1:100, Thermo Scientific, PA5-47488, https://www.thermofisher.com/antibody/product/ACE2-Antibody-Polyclonal/PA5-47488), rabbit anti-lysozyme (1:500, Thermo Scientific, MA5-32154, Clone ST50-02, https://www.thermofisher.com/antibody/product/Lysozyme-Antibody-clone-ST50-02-Recombinant-Monoclonal/MA5-32154), rabbit anti-BrdU (1:100, Abcam, ab6326, clone BU1/75 (ICR1), https://www.abcam.com/en-us/products/primary-antibodies/brdu-antibody-bu1-75-icr1-proliferation-marker-ab6326), rabbit anti–Cl. Caspase 3 (p 175) (1:200, Cell Signaling, 9664S, clone 5A1E, https://www.cellsignal.com/products/primary-antibodies/cleaved-caspase-3-asp175-5a1e-rabbit-mab/9664), mouse anti-Hnf4a (1:100, Thermo Scientific, MA1-199, clone K9218, https://www.thermofisher.com/antibody/product/HNF4A-Antibody-clone-K9218-Monoclonal/MA1-199), mouse anti-Tet1 (1:100, Thermo Scientific, MA5-16312, clone GT1462, https://www.thermofisher.com/antibody/product/TET1-Antibody-clone-GT1462-Monoclonal/MA5-16312), rabbit anti-Tet2 (1:100, Thermo Scientific, PA5-85488, https://www.thermofisher.com/antibody/product/TET2-Antibody-Polyclonal/PA5-85488), rabbit anti-Tet3, (1:100, Thermo Scientific, PA5-31860, https://www.thermofisher.com/antibody/product/TET3-Antibody-Polyclonal/PA5-31860), rat anti-Cd8 (1:200, 14-0808-82, Thermo Scientific, Clone 4SM15, https://www.thermofisher.com/antibody/product/CD8a-Antibody-clone-4SM15-Monoclonal/14-0808-82) rat anti-Cd4 (1:100, Thermo Scientific, 14-9766-82, Clone 4SM95, https://www.thermofisher.com/antibody/product/CD4-Antibody-clone-4SM95-Monoclonal/14-9766-82). Primary antibodies were detected with the following fluorescently conjugated secondary antibodies: goat anti-chicken AF488 (1:1000, Life Technologies A-11039), goat anti-rabbit AF488 (1:1000, Life Technologies A-32723), goat anti-rabbit AF594 (1:1000, Life Technologies A-11037), goat anti-mouse AF488 (1:1000, Life Technologies, A-32723), goat anti-mouse AF594 (1:1000, Life Technologies, A-11032), goat anti-rat AF488 (1:1000, Life Technologies, A-11006) and goat anti-rat 594 (1:1000, Life Technologies, A-11007).
The antibodies used in COMET experiments are: Atho1 (anti-Rabbit, 21215-1-AP, Proteintech, 1:100, https://www.ptglab.com/results?category=&q=21215-1-AP&target=), Cd3 (anti-Rabbit, ab5690, Abcam, 1:100, https://www.abcam.com/en-us/products/primary-antibodies/cd3-epsilon-antibody-ab5690), Sma (anti-Rabbit, ab5694, Abcam, 1:400, https://www.abcam.com/en-us/products/primary-antibodies/alpha-smooth-muscle-actin-antibody-ab5694), F480 (anti-Rabbit, 7006, Clone D2S9R, Cell Signaling, 1:100, https://www.cellsignal.com/products/primary-antibodies/f4-80-d2s9r-xp-rabbit-mab/70076), Ogdh (anti-Rabbit, 15212-1-AP, Proteintech, 1:200, https://www.ptglab.com/products/OGDH-Antibody-15212-1-AP.htm?srsltid=AfmBOoohrwP797YeCUvojyQLmEek1jzAUChFjXOtfu6_Gs32qL0SiaKC), 5hmC (anti-Rabbit, 39769, Active Motif, 1:150, https://www.activemotif.com/catalog/details/39769), CC3 (Ser 175) (Cell Signaling, 9664S, clone 5A1E, https://www.cellsignal.com/products/primary-antibodies/cleaved-caspase-3-asp175-5a1e-rabbit-mab/9664), MPO (anti-Rabbit, Ab9535, Abcam, 1:150, https://www.abcam.com/en-us/products/primary-antibodies/myeloperoxidase-antibody-ab9535), Ephb2 (anti-Rabbit, ab252935, Abcam, 1:100, Clone EPR22427-268, https://www.abcam.com/en-us/products/primary-antibodies/eph-receptor-b2-antibody-epr22427-268-ab252935), Cd31 (anti-Rabbit, ab28364, Abcam, 1:50, https://www.abcam.com/en-us/products/primary-antibodies/cd31-antibody-

ab28364), p53 (anti-Rabbit, NCL-L-p53-CM5p, Leica Biosystems, 1:100 https://shop.leicabiosystems.com/es-es/ihc-ish/ihc-primary-antibodies/pid-p53-protein-cm5), Muc2 (anti-Rabbit, PA5-21329, Invitrogen, 1:500, https://www.thermofisher.com/antibody/product/MUC2-Antibody-Polyclonal/PA5-21329), Axin2 (anti-Rabbit, ab32197, Abcam, 1:100, https://www.abcam.com/en-us/products/primary-antibodies/axin-2-antibody-ab32197), Ki67 (anti-Mouse, BD, 550609, Clone 56 (RUO), 1:100, https://www.bdbiosciences.com/en-es/products/reagents/flow-cytometry-reagents/research-reagents/single-color-antibodies-ruo/purified-mouse-anti-ki-67.550609?tab=product_details), Cd4 (anti-Rat, 4SM95, eBiosciences, 1:100, https://www.thermofisher.com/antibody/product/CD4-Antibody-clone-4SM95-Monoclonal/14-9766-82), B220 (anti-Rat, Clone RA3-6B2, BioLegend, 1:100, https://www.biolegend.com/en-us/products/purified-anti-mouse-human-cd45r-b220-antibody-449), PanCK (anti-Mouse, AE1/AE3, Abcam, 1:50, https://www.abcam.com/en-us/products/primary-antibodies/pan-cytokeratin-antibody-ae1-ae3-5d3-ab86734), Cd8 (anti-Rat, 14-0808-82, Thermo Scientific, Clone 4SM15, 1:100, https://www.thermofisher.com/antibody/product/CD8a-Antibody-clone-4SM15-Monoclonal/14-0808-82), bCat (anti-Mouse, BD, 610153, clone 14/ catenin (RUO), 1:100, https://www.bdbiosciences.com/en-es/products/reagents/microscopy-imaging-reagents/immunofluorescence-reagents/purified-mouse-anti-catenin.610153?tab=product_details), and LMNB1 (anti-Mouse, clone a11, sc-377000, Santacruz, 1:300, https://www.scbt.com/es/p/lamin-b1-antibody-a-11). For more details, check Supplementary Table 4.
The antibodies used in COMET experiments are: aSMA (anti-Rabbit, Cell Signaling CST, 34105S, D4K9N, 1:50, https://www.cellsignal.com/products/antibody-conjugates/a-smooth-muscle-actin-d4k9n-xp-rabbit-mab-alexa-fluor-488-conjugate/34105), CD3Ɛ (anti-Rabbit, Cell Signaling CST, 57869BC, D7A6E, 1:100, https://www.cellsignal.com/products/antibody-conjugates/cd3e-d7a6e-xp-rabbit-mab-alexa-fluor-555-conjugate/57869), CD163 (anti-Rabbit, Cell Signaling CST, 39093BC, D6U1J, 1:100, https://www.cellsignal.com/products/antibody-conjugates/cd163-d6u1j-rabbit-mab-alexa-fluor-647-conjugate/39093), Vimentin (anti-Rat, R&D Systems, #280618, IC2105S, 1:300, https://www.rndsystems.com/products/human-mouse-rat-vimentin-antibody-280618_mab2105), Hnf4a (anti-Rabbit, CST, 31059, C11F12, 1:100, https://www.cellsignal.com/products/primary-antibodies/hnf4a-c11f12-rabbit-mab-bsa-and-azide-free/31059), Muc2 (anti-Rabbit, PA5-21329, Invitrogen, 1:500, https://www.thermofisher.com/antibody/product/MUC2-Antibody-Polyclonal/PA5-21329), CD8A (anti-Rabbit, Cell Signaling CST, 77909S, D8A8Y, 1:100, https://www.cellsignal.com/products/antibody-conjugates/cd8a-d8a8y-rabbit-mab-alexa-fluor-555-conjugate/77909), CD45 (anti-Rabbit, CST, 19744S, D9M8I, 1:50, https://www.cellsignal.com/products/antibody-conjugates/cd45-intracellular-domain-d9m8i-xp-rabbit-mab-alexa-fluor-647-conjugate/19744), CD31 (Pecam-1) (anti-Mouse, Cell Signaling CST, 61255S, 89C2, 1:50, https://www.cellsignal.com/products/antibody-conjugates/cd31-pecam-1-89c2-mouse-mab-alexa-fluor-555-conjugate/61255), CD56 (anti-Rabbit, Cell Signaling CST, 50831BC, E7X9M, 1:100, https://www.cellsignal.com/products/antibody-conjugates/ncam1-cd56-e7x9m-xp-rabbit-mab-alexa-fluor-647-conjugate/50831), CC3 (anti-Rabbit, Cell Signaling CST, 97774S, D3E9, 1:100, https://www.cellsignal.com/products/antibody-conjugates/cleaved-caspase-3-asp175-d3e9-rabbit-mab-alexa-fluor-750-conjugate/97774), PANCK (anti-Mouse, ThermoFisher, 53-9003-82, AE1/AE3, 1:100, https://www.thermofisher.com/antibody/product/Pan-Cytokeratin-Antibody-clone-AE1-AE3-Monoclonal/53-9003-82), CD68 (anti-Rabbit, Cell Signaling CST, 23308S, D4B9C, 1:50, https://www.cellsignal.com/products/antibody-conjugates/cd68-d4b9c-xp-rabbit-mab-alexa-fluor-555-conjugate/23308), CD11B (anti-Rabbit, Cell Signaling CST, 79750S, D6X1N, 1:100, https://www.cellsignal.com/products/antibody-conjugates/cd11b-itgam-d6x1n-rabbit-mab-alexa-fluor-647-conjugate/79750), MPO (anti-Goat, R&D Systems, AF3667, 1:100, https://www.rndsystems.com/products/human-mouse-myeloperoxidase-mpo-antibody_af3667), Cd11c (anti-Hamster, Invitrogen, 58-0114-80, N418, 1:100, https://www.thermofisher.com/antibody/product/CD11c-Antibody-clone-N418-Monoclonal/58-0114-82), p53 (anti-Mouse, BD, DO-1, 1:100), Ki67 (anti-Rabbit, BD Pharmingen, 558617, B56, 1:50, https://www.bdbiosciences.com/en-es/products/reagents/flow-cytometry-reagents/research-reagents/single-color-antibodies-ruo/purified-mouse-anti-human-p53.554294?tab=product_details), Ogdh (anti-Rabbit, 15212-1-AP, Proteintech, 1:200, https://www.ptglab.com/products/OGDH-Antibody-15212-1-AP.htm?srsltid=AfmBOoohrwP797YeCUvojyQLmEek1jzAUChFjXOtfu6_Gs32qL0SiaKC), Beta-Catenin (anti-Mouse, BD, 610153, clone 14/ catenin (RUO), 1:100, https://www.bdbiosciences.com/en-es/products/reagents/microscopy-imaging-reagents/immunofluorescence-reagents/purified-mouse-anti-catenin.610153?tab=product_details), cd4 (anti-Rat, ThermoFisher, 14-9766-82, 4SM95, 1:100, https://www.thermofisher.com/antibody/product/CD4-Antibody-clone-4SM95-Monoclonal/14-9766-82), 5hmC (anti-Rabbit, 39769, Active Motif, 1:50, https://www.activemotif.com/catalog/details/39769), gH2AX (anti-Mouse, Sigma, 05-636, JBW301, 1:100, https://www.sigmaaldrich.com/ES/es/product/mm/05636af647).

AF700 CD45 (BioLegend, 103128, Clone 30-F11, 1:200, https://www.biolegend.com/en-us/products/alexa-fluor-700-anti-mouse-cd45-antibody-3407), BUV395 CD11b (BD Biosciences, 563553, Clone M1/70, 1:200, https://www.bdbiosciences.com/en-us/search-results?searchKey=563553), PE F4/80 (BioLegend, 123110, Clone BM8, 1:200, https://www.biolegend.com/en-ie/products/pe-anti-mouse-f4-80-antibody-4068), BV605 Ly6G (BD Bioscience, 563005, Clone 1A8, 1:200, https://www.bdbiosciences.com/en-es/products/reagents/flow-cytometry-reagents/research-reagents/single-color-antibodies-ruo/bv605-rat-anti-mouse-ly-6g.563005?tab=product_details), APC Cy7 Ly6c (BioLegend, 128026, Clone HK1.4, 1:200, https://www.biolegend.com/en-ie/products/apc-cyanine7-anti-mouse-ly-6c-antibody-6758), APC MHCII (BioLegend, 107614, Clone M5/114.15.2, 1:200, https://www.biolegend.com/en-ie/products/apc-anti-mouse-i-a-i-e-antibody-2488), BV710 CD206 (BioLegend, 141727, Clone C068C2, 1:200, https://www.biolegend.com/en-ie/products/brilliant-violet-711-anti-mouse-cd206-mmr-antibody-12012), BV650 CD86 (BioLegend, 105035, GL-1, 1:200, https://www.biolegend.com/en-ie/products/brilliant-violet-650-anti-mouse-cd86-antibody-7643).

Validation

All antibodies were commercially available and were validated by the provider. See the links above.

Animals and other research organisms

Policy information about studies involving animals; ARRIVE guidelines recommended for reporting animal research, and Sex and Gender in Research

Laboratory animals

C57Bl/6J mice or genetically engineered mouse models (backcrossed to a Bl6 background) were used in this study.All animal experiments in this study were performed in accordance with protocols approved by the Memorial Sloan Kettering Institutional Animal Care and Use Committee (approval number: 11-06-012). The mice were housed with a 12 h light/dark cycle between 8:00 and 20:00 in a temperature-controlled room (22 ± 1°C) with free access to water and food. Both male and female mice were used in equal proportions for all experiments. No sex-based differences were observed. Experiments were performed using mice aged 10 to 14 weeks. Sample sizes were determined based on prior experiments and published studies to ensure adequate power to detect

	biologically relevant differences. Mice were randomly assigned to experimental groups as reported before. Investigators were blinded to group allocation during data collection and analysis whenever possible.
Wild animals	The study did not involved wild animals
Reporting on sex	The experiments described in the manuscript were conducted on both sexes, including both female and male mice in the results
Field-collected samples	The study did not involved field collected samples
Ethics oversight	All animal experiments in this study were performed in accordance with protocols approved by the Memorial Sloan Kettering Institutional Animal Care and Use Committee (approval number: 11-06-012).

Note that full information on the approval of the study protocol must also be provided in the manuscript.

Plants

Seed stocks	N/A
Novel plant genotypes	N/A
Authentication	N/A

Flow Cytometry

Plots

Confirm that:

☒ The axis labels state the marker and fluorochrome used (e.g. CD4-FITC).

☒ The axis scales are clearly visible. Include numbers along axes only for bottom left plot of group (a 'group' is an analysis of identical markers).

☒ All plots are contour plots with outliers or pseudocolor plots.

☒ A numerical value for number of cells or percentage (with statistics) is provided.

Methodology

Sample preparation	-Generation of an immune-mediated colitis model (Rag2-/- mice): To generate a rodent model of human IBD, we used the CD4+CD45RBHigh-induced colitis model in Rag2-/- mice44. Briefly, spleens from 10 C57Bl/6 male mice were collected, smashed, and filtered through a 40- m filter and washed with isolation buffer (PBS, 0.5% bovine serum albumin (BSA) and 2 mM EDTA, pH = 7.2). The cells were then centrifuged (288g , 5 min), and the resulting splenocyte pellets were resuspended in ACK buffer (Quality Biologicals, 118-156-101CS) to lyse red blood cells. After cell counting, CD4+ cells were isolated using the CD4+ isolation kit (Miltenyi, 130-104-454) following the manufacturer's instructions. The splenocytes were transferred to FACS buffer (0.5% BSA and 2 mM EDTA in Ca2+/Mg2+-free PBS) and incubated on ice for 30 minutes with anti-CD4-APC (BioLegend, 116014, Clone RM4-4) and anti-CD45Rb-FITC (BioLegend, 103306, Clone: C363-16A) antibodies. CD4 +CD45RbHigh and CD4+CD45RbLow cells were then sorted using a Sony MA900 cell sorter.<br><br>- uFor organoids: Organoids were harvested from Matrigel using Cell Recovery Solution and subsequently incubated in TriplE for 3 minutes at 37°C. Following this, they were washed and resuspended in FACS buffer before cell sorting.<br><br>-Immunophenotyping in DSS-treated mice: Enrichment in the immune fraction as performed as previously descrined (https://www.nature.com/articles/nprot.2007.315). For multi-parametric flow cytometry analysis, cell suspensions were stained with LIVE/DEAD fixable viability dye (1: 500, Invitrogen, R37601) for 30 min in PBS at 4°C. After this, cells were washed, incubated with Fc block (1: 200, BD Bioscience, 564219) in FACS buffer for 15 min at 4°C, and then stained with a cocktail of conjugated antibodies (see below) for 30 min on ice. After staining, cells were washed 3 times with FACS buffer and fixed using BD Cytofix/ Cytoperm (Fisher Scientific, 544772) for 20 min at 4 °C, washed again, and stored for analysis. Samples were analyzed in a BD LSR Fortessa with 5 lasers, where gates were set by use of fluorescence-minus-one (FMO) controls.
Instrument	Sorting experiments were performed in Sony MA900 cell sorter. Flow-based analysis was done in Fortessa II cytometre.
Software	FlowJo was used to analyze the data
Cell population abundance	Immune cell infiltration was determined by first gating in the Cd45+ cells and then, drilling down from there.
Gating strategy	In our experiments, we employed a comprehensive gating strategy starting with preliminary forward scatter (FSC) and side scatter (SSC) gating to identify the initial cell population. After gating on the initial FSC-A vs SSC-A plot to include all events of

interest (cells), we utilized subsequent plots such as FSC-A vs FSC-H or SSC-A vs SSC-W to further refine our analysis. Doublets typically exhibit higher FSC-A or SSC-A values relative to their FSC-H or SSC-W values compared to single cells, resulting in distinct cloud or diagonal patterns in these plots. This approach ensures accurate identification and exclusion of cell aggregates, maintaining the integrity of our flow cytometry data. Subsequently, cells were gated based on live/dead dyes, and only the live population (negative for live/dead dyes) was subjected to further analysis or sorting.

In the organoid experiments, we sorted RFP+ viable cells. In Rag-/- mice, we sorted CD4+CD45RBhigh or CD4+CD45RBlow cells. For immune phenotyping, we gated on the CD45+ populations and subsequently analyzed the immune composition based on various markers.

☒ Tick this box to confirm that a figure exemplifying the gating strategy is provided in the Supplementary Information.
